# Confirmatory reinforcement learning changes with age during adolescence

**DOI:** 10.1111/desc.13330

**Published:** 2022-10-27

**Authors:** Gabriele Chierchia, Magdaléna Soukupová, Emma J. Kilford, Cait Griffin, Jovita Leung, Stefano Palminteri, Sarah-Jayne Blakemore

**Affiliations:** a Department of Psychology, University of Cambridge, UK; b Institute of Cognitive Neuroscience, University College London, UK; c Department of Cognitive Science, École Normale Supérieure, FR; d Department of Clinical, Educational and Health Psychology, University College London, UK; e Institute of Cognitive Neuroscience, HSE, Moscow, Federation of Russia

**Keywords:** Confirmation bias, reinforcement learning, adolescence, exploration, computational modelling, learning rates

## Abstract

Understanding how learning changes during human development has been one of the long-standing objectives of developmental science. Recently, advances in computational biology have demonstrated that humans display a bias when learning to navigate novel environments through rewards and punishments: they learn more from outcomes that confirm their expectations than from outcomes that disconfirm them. Here, we ask whether confirmatory learning is stable across development, or whether it might be attenuated in developmental stages in which exploration is beneficial, such as in adolescence. In a reinforcement learning task, 77 participants aged 11-32 years (4 men, mean age = 16.26) attempted to maximize monetary rewards by repeatedly sampling different pairs of novel options, which varied in their reward/punishment probabilities. Mixed-effect models showed an age-related increase in accuracy as long as learning contingencies remained stable across trials, but less so when they reversed halfway through the trials. Age was also associated with a greater tendency to *stay* with an option that had just delivered a reward, more than to *switch* away from an option that had just delivered a punishment. At the computational level, a confirmation model provided increasingly better fit with age. This model showed that age differences are captured by decreases in noise or exploration, rather than in the magnitude of the confirmation bias. These findings provide new insights into how learning changes during development and could help better tailor learning environments to people of different ages.

## Introduction

Confirmation biases involve the tendency to assign greater weight to confirmatory than disconfirmatory evidence (Nickerson, 1998). Confirmation biases are amongst the most well researched biases in cognitive science (Benjamin, 2019) and have been shown to affect social judgements (Snyder et al., 1978), investments (Park et al., 2013), medical diagnoses (Mendel et al., 2011) and information bubbles (Knobloch-Westerwick et al., 2020), among many other domains. Recently, computational approaches have demonstrated that a form of confirmation bias can also affect how an individual’s behaviour is shaped by rewards and punishments, one of the building blocks of learning, known as reinforcement learning (RL). Indeed, studies have shown that, as agents learn to navigate novel environments through reward and punishment, they tend to learn more from outcomes that confirm their choices, than from outcomes that disconfirm their choices, a phenomenon called *choice confirmation bias, or confirmatory learning* (Palminteri & Lebreton, 2022 for a review). Choice confirmation bias has been observed in human adults (Palminteri et al., 2017), adolescents (e.g., Nussenbaum et al., 2021; Xia et al., 2021) and children (Habicht et al., 2021), as well as rodents (Ohta et al., 2021) and monkeys (Farashahi et al., 2019), and in many different learning environments (Lefebvre et al., 2022; Tarantola et al., 2021). Despite these advances, the developmental trajectories of confirmatory RL remain unclear. It is possible that confirmatory RL is attenuated during stages of development in which more exploratory learning styles are beneficial, such as adolescence. The current study aimed to address this proposal.

Adolescence, defined as the age between 10 and 24 (Sawyer et al., 2018), is considered a sensitive period of development (Fuhrmann et al., 2015; Laube et al., 2020), in which developmental changes in the brain and the mind enable and motivate individuals to become independent from their caregivers, by exploring new activities and social environments as opposed to confirming (building upon) pre-existing ones. In addition, adolescents have less experience than adults on which to base their beliefs, preferences and confirmation biases (in Bayesian terminology, they have broader priors) (Tenenbaum et al., 2011). For example, relative to adults, adolescents tend to be less certain about their preferences (Reiter et al., 2021), display more variance in their choices (Ciranka & van den Bos, 2021; Martin et al., 2018) and are more tolerant to making decisions under ambiguity (Tymula et al., 2012; van den Bos & Hertwig, 2017). In parallel, across cultures, sensation seeking shows a quadratic developmental trajectory, increasing in the teenage years and peaking in the late teens, then decreasing in early adulthood (Steinberg et al., 2018). Other learning-related processes, such as non-verbal reasoning (Chierchia et al., 2019) and inhibitory control (Constantinidis & Luna, 2019), also develop during adolescence, though more linearly or asymptotically. These findings are consistent with the notion that adolescence is characterised by a transition between more exploratory to more confirmatory learning styles (Conway, 2020; Frankenhuis & Walasek, 2020; Giron et al., 2022; Gopnik, 2020).

In line with this, studies have demonstrated that adults are less exploratory or less ‘noisy’ in their RL behaviour than adolescents aged 12 - 18 years (Bolenz et al., 2017; Nussenbaum & Hartley, 2019, for reviews). Frequently, this has been associated with the notion that adolescents generally perform worse than adults in RL. However, and in contrast to this, recent studies have also pointed to possible advantages of heightened exploration during adolescence. These studies suggest that such adolescent advantages can particularly emerge in more volatile environments in which flexibility is more useful (Eckstein et al., 2022; Lloyd et al., 2021). This raises the question of whether such age-related differences in RL could be explained by changes in confirmatory learning. To assess this, we investigated whether certain behavioural patterns of confirmatory learning become more pronounced with age between early adolescence and early adulthood. In particular, previous work (Lefebvre et al., 2022; Palminteri et al., 2017) has shown that, in adults, confirmation bias is associated with three behavioural patterns that can be detected through three different RL environments. In addition to these, we propose a fourth behavioural pattern, which is associated with confirmatory learning across these environments. Below we illustrate these patterns of confirmatory learning, at the behavioural and computational levels.

### Confirmatory reinforcement learning - behavioural level

First, choice confirmation bias predicts better performance in a number of RL environments (Chambon et al., 2020; Lefebvre et al., 2022; Tarantola et al., 2021). While this sounds counterintuitive, it has been suggested that psychologically inflating confirmatory evidence can serve to buffer the impact of noise on decision making (Lefebvre et al., 2022; Qiu et al., 2020), possibly preserving against excessive self-doubt or uncertainty. In particular, simulations suggest that confirmation bias is especially advantageous in certain stable (henceforth *stationary*) learning environments (Lefebvre et al., 2022), that is, environments in which learning contingencies do not change. This could occur because in stationary learning environments past outcomes (i.e., the evidence history) are, by definition, more predictive of future outcomes and should allow people to generate more reliable priors (one’s current belief about the probability distribution of future outcomes before any further evidence is sampled). Over-emphasizing outcomes that confirm one’s choices could therefore be adaptive if those outcomes are noisy or probabilistic but stationary. The hypothesis that confirmatory learning increases with age during adolescence is consistent with the frequently observed positive association between age and RL accuracy (see Bolenz et al., 2017 and Nussenbaum & Hartley, 2019, for reviews).

Second, by the same reasoning, the advantages of confirmation bias on performance are reduced when learning contingencies change, that is, in more volatile environments (Lefebvre et al., 2022; Palminteri et al., 2017). In such cases, confirmatory learning could instead serve to momentarily decrease performance. For example, if a previously advantageous option becomes suddenly disadvantageous, a higher confirmation bias could lead one to initially discount the disconfirmatory evidence incurred when contingencies change, leading to some perseveration in selecting options that are no longer advantageous (e.g., a greater lag in reversal learning). In other words, while confirmatory bias can lead to faster learning in stationary learning environments that can result in greater overall performance, it can also result in a greater negative impact (or ‘harder crash’) on performance when contingencies change. This prediction is consistent with recent findings that age-related benefits are largely reduced, and can be even reversed, in more volatile environments (Eckstein et al., 2022; Lloyd et al., 2021).

Third, confirmation bias can result in biased sampling behaviours in situations in which neither option is better than the other. This case is peculiar because if no option is best (e.g., each option is equally likely to deliver a reward/punishment), there is no objective way to assess performance. However, this condition potentially allows the observation of a different confirmation bias signature: people should develop a tendency to select *one* of the options more frequently than the others, even if the evidence does not discriminate between options. In contrast, unbiased learners would sample equally advantageous options to similar extents. In other words, if adults assign more weight to confirmatory than disconfirmatory evidence, they might tend to choose one option more frequently than the other, *as if* that option had been rewarded more often (i.e., confirmed). Adolescents might, on the other hand, show a more unbiased selection of the options, thus more closely aligning their behaviour with the evidence they experienced. We refer to this behavioural pattern as choice conservatism. The link between confirmatory learning and conservatism has been observed in adults (Palminteri et al., 2017) but has not yet, to our knowledge, been investigated in adolescents.

Fourth, confirmation bias can be associated with an amplified win-stay/lose-shift asymmetry. Most learning rules suggest that winning should increase the probability of staying with a given option, while losing should induce switching away from it (Sutton & Barto, 1998). As learning progresses however, both biased and unbiased learners should begin to show an asymmetry in the frequency of these two behaviours: they should stay after wins more than they shift after losses. This is because if someone has learned that an option is generally better than another, they should continue to choose it even when it occasionally delivers a loss. However, if people place higher weight on confirmatory evidence than disconfirmatory evidence, they might show an amplified asymmetry in such win-stay/lose-shift behaviour. More specifically, we hypothesized that the win-stay/lose-shift asymmetry should increase with age. This prediction has been supported by previous work showing that, relative to adolescents, adults are more likely to learn from positive outcomes than from negative outcomes (Hauser et al., 2015; Rosenbaum et al., 2022; van den Bos et al., 2012; Xia et al., 2021; see Nussenbaum & Hartley, 2019 for a discussion).

In addition to addressing how these four decision patterns of confirmatory learning change with age, as secondary exploratory variables, we assessed decision times and outcome observation times. Given the evidence that impulsivity is heightened in adolescence (Steinberg, 2010; Ziegler et al., 2019), one possible explanation for age-related improvements in accuracy could be that younger participants spend too little time making decisions and learning from their outcomes. Controlling for decision times and outcome observation times will help address whether age-related reductions in accuracy can be partly explained by a reduction in impulsivity or increases in attention.

### Confirmatory reinforcement learning – computational level

Computationally, choice confirmation bias is captured by an asymmetry in learning rates. Learning rates are behaviourally estimated parameters that regulate the extent to which agents update option values when encountering a discrepancy between expected and experienced outcomes (a prediction error). Prediction errors can be positive (better than expected) or negative (worse than expected), and traditional RL models assume that agents do not distinguish between the two (Sutton & Barto, 1998). In contrast, RL models that allow positive and negative learning rates to vary separately have been found to better account for observed behaviour (Palminteri & Lebreton, 2022). In addition, positive learning rates are frequently larger than negative ones, and the extent of this discrepancy can be used as a measure of choice confirmation bias.

Importantly, the four age-related behavioural patterns described above are not exclusively consistent with confirmatory learning. We illustrate this by focussing on the first of the anticipated patterns: the age-related improvement in RL accuracy. Even a traditional RL model could account for this by assigning more optimal learning rates to adults than adolescents (Decker et al., 2015; Master et al., 2020). Alternatively, different learning models might apply to people of different ages, supporting qualitative rather than quantitative differences in learning styles between adults and adolescents. For example, one study found that adolescent RL was best described by a model with a single learning rate that was only shaped by the outcomes of chosen options, and not by the outcomes of unchosen options (which were nonetheless observed). In contrast, adult RL was captured by more sophisticated models that also tracked the outcomes of unchosen options (i.e., models that incorporated counterfactual evidence) (Palminteri et al., 2016). An even simpler model than Q-learning is a random model (Wilson & Collins, 2019), which assumes that people randomize their choices between options, but might have a random bias towards one of them. This model assumes that participants are insensitive to option values. Nonetheless, even a random model could still pick up on age-related differences in RL accuracy, for example, by estimating greater option biases in adults (which happen to be in favour of the correct option).

In addition to learning rates, which regulate how values are learned, common computational models connect these values to choices through another parameter called inverse temperature (Equation 4). When inverse temperature is high, even a small difference in option values is sufficient to direct choice, whereas people with lower inverse temperature will more frequently choose sub-optimal options. The term derives from sciences on solids, such as metals, which are more flexible at higher temperatures, and has been used to describe certain statistical optimization algorithms (Kirkpatrick et al., 1983). These algorithms typically begin by performing large and unpredictable shifts in parameter adjustment. As they approach an optimal solution, they cool off: their adjustments become smaller and more predictable. Intriguingly, recent accounts have suggested that this “cooling off” metaphor well describes how learning changes with age across development (Giron et al., 2022; Gopnik, 2020). Indeed, inverse temperature has frequently been found to increase between adolescence and adulthood, providing a plausible computational basis to age-related benefits in learning (Nussenbaum & Hartley, 2019 for a review).

In summary, different models, differential model fits, or different parameter settings within models (e.g., related to learning rate asymmetries or inverse temperature), could theoretically be consistent with the first behavioural pattern of interest: a commonly observed positive trend between age and RL accuracy. An optimal RL model should be able to explain the other three predicted age-related trends as well, and to do so better than alternative models. Thus, together with the four age related patterns illustrated above, here, we aim to characterize the computational source of age-related differences in learning between adolescence and adulthood.

### The current study

In the current study, we addressed the question of whether confirmatory learning increases with age between adolescence and adulthood. We address this behaviourally and computationally, by employing a standard RL task in which participants between the ages of 11 and 32 years repeatedly chose between fixed pairs of options (pairs of novel symbols) that varied in their monetary reward/punishment probabilities. At the behavioural level, in line with the four decision patterns illustrated above, we hypothesised that, if confirmatory learning increases with age between adolescence and adulthood, increasing age should be associated with different behaviours in different environments. Specifically, we made four predictions that there would be: P1) increased accuracy in stationary and asymmetric conditions (where one option is more advantageous than the other); P2) reduced age-related advantages in reversal learning conditions; P3) increased likelihood of preferring one option over another (choice conservatism) when both are equally advantageous, namely, in stationary but symmetric conditions; and P4) increased win-stay/lose-shift asymmetry across conditions. At the computational level, we predicted (P5) that these age trends would be best captured by a confirmation model, which allows confirmatory and disconfirmatory learning rates to vary independently.

## Methods

### Participants

We recruited 77 participants (4 men, age range 11-32 years; *M* = 16.26, *SD* = 4.62). Our sample size was determined based on previous computational studies, which observed developmental trends in RL with sample sizes ranging between 50 and 100 (Cohen et al., 2020; Habicht et al., 2021; Palminteri et al., 2016; Rosenbaum et al., 2022) (see Supplementary Material 1 for a power analysis). The much larger prevalence of women/girls in our sample was due to logistic constraints of school testing. Given there were only four men and that they were all over 18 years of age, modelling interactions between age and gender was not feasible. Instead, to not sacrifice any power, we conducted our main analyses on all participants and then conducted sensitivity analyses by re-running all models on women/girls only. The results were unchanged unless otherwise noted. To avoid arbitrary age-related grouping criteria, key analyses employed age as a continuous variable. However, for illustrative purposes only, we also present data dividing our sample into “adults” (N = 20, age range 18-32 years; M = 22.85, SD = 3.09), and “adolescents” (N = 57, age range 11-17; M = 13.95, SD = 2.15). The data from adult participants has been published previously (Palminteri et al., 2017). All participants received £5 for their participation, plus up to £15, proportionally to the points accumulated during the task. Adult participants, and parents of participants under 18 years, gave written informed consent and the study was approved by the UCL ethics committee (number: 3453/001).

### Experimental task

**Fig. 1:**
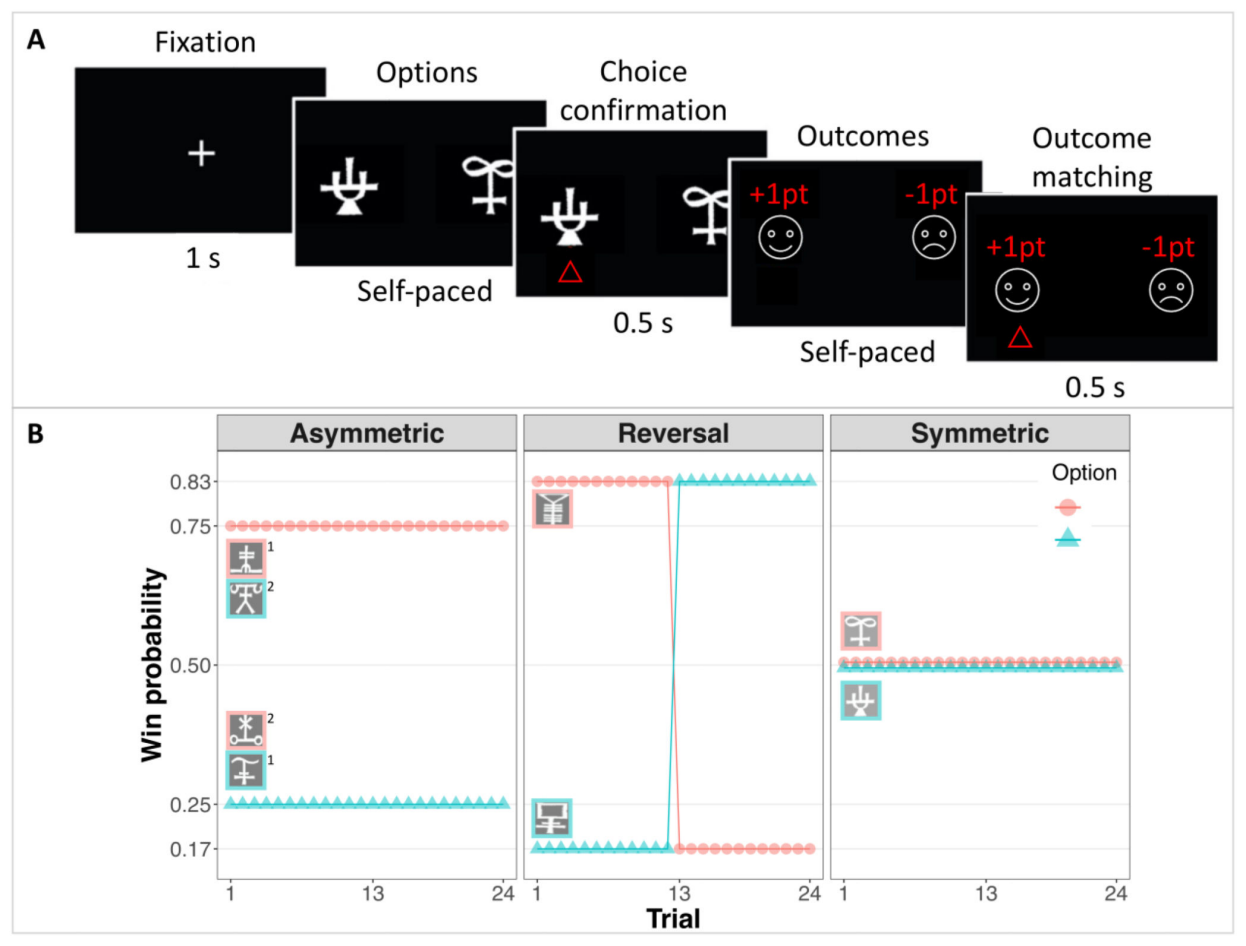
**A. Trial structure**. After a fixation cross, participants chose between one of two symbols by pressing one of two keys and their choice was confirmed by the appearance of a red arrow under the chosen option. The outcomes of both the chosen and the unchosen options were then revealed. To move on to the next trial, participants had to press the same key as before, thus matching the outcome. Decision times and outcome observation times were self-paced. **B. Experimental conditions**. Participants were told that some symbols might lead to winning more often than others. They were asked to try to discover which option was more advantageous by trial and error. Symbols were presented in fixed pairs, for 24 trials. There were 4 pairs of symbols in three conditions. In the asymmetric condition, one symbol led to winning more frequently than the other throughout the session. In the reversal condition, one symbol led to winning more frequently than the other for the first half of the trials, but these contingencies reversed in the second half. In the symmetric condition, both symbols were equally advantageous. The asymmetric condition was repeated twice, each time with a new pair of symbols. The reversal and symmetric conditions each occurred once, each time with a unique pair of symbols. The exact probabilities of winning 1 point for each option are shown on the y-axis, for each condition (losing occurred with the complimentary probability), as function of the trial. Symbol pairs were presented in an interleaved fashion.

Participants took part in a standard probabilistic instrumental learning task (Fig. 1). On multiple trials, they were asked to choose one of two visual stimuli (Fig. 1 Panel A), resulting in two possible outcomes: winning 1 point or losing 1 point. Participants were encouraged to accumulate as many points as possible and were informed that some stimuli would result in winning more often than others. Participants were given no explicit information regarding these reward probabilities. Instead, they had to learn these through trial and error. To allow learning to occur, the same two stimuli were presented in fixed pairs for 24 trials. On each trial (Fig. 1, Panel A), after a 1 s fixation cross, the two stimuli were presented. Participants made their decisions by pressing left or right arrow keys with their right hand. The decision time was self-paced, recorded and analysed. Decisions were confirmed by the appearance of a red triangle under the chosen option, lasting 0.5 s. Outcomes for both the chosen and unchosen option were then shown. The obtained outcomes were presented in the same place as the chosen stimulus and the forgone outcomes in the same place as the unchosen stimulus. To move to the subsequent trial, participants had to match the position of the chosen outcome with a key press (right/left arrow). These outcome observation times were also self-paced and analysed. There were 4 pairs of stimuli, presented in a pseudo-randomly interleaved fashion. For each pair, the reward probabilities of each of the stimuli varied according to three experimental conditions described below (Fig. 1, Panel B).

In the *asymmetric* condition, one stimulus was consistently more likely to result in a win than the other. Specifically, the probabilities of winning 1 point were 0.75 for one option and 0.25 for the other, and these probabilities remained stationary for this pair of stimuli. In the *reversal* condition, one stimulus was also better than the other, but only in the first half of the block (the first 12 trials). In the second half of the block (trials 13 to 24), these probabilities were reversed, such that the previously advantageous option become disadvantageous and vice versa. Specifically, in the pre-reversal portion of the reversal condition, the probabilities of winning 1 point were 0.83 for one stimulus, and 0.17 for the other, and these probabilities reversed in the post-reversal portion of the condition. These reward probabilities were slightly modified relative to the asymmetric condition to compensate for the fact that, in the reversal condition, participants had fewer trials to learn (12 trials vs. 24). Finally, in the *symmetric* condition both options were equally likely to result in a win/loss, with a probability of 0.5. These three conditions respectively allow to address the three predictions introduced above: P1) Age would improve accuracy in stationary environments (asymmetric condition); P2) Age-related benefits would decrease in volatile environments (reversal condition); P3) Age would be associated with an increased tendency to choose the same option (choice conservatism) when both options are equally advantageous (symmetric condition); P4) Across conditions, age should be associated with an increased tendency to stay after wins as opposed to shift after losses.

Overall, the 4 pairs of 24 trials amounted to 96 trials across the session. Each fixed pair of stimuli was used in one condition only, except for the asymmetric condition, which employed two pairs of stimuli. We emphasized the asymmetric condition for consistency with the previous study from which the adult sample was drawn (Palminteri et al., 2017). To account for the power imbalance between conditions that resulted from this, we conducted sensitivity analyses by re-running all our main analyses twice: once without the first asymmetric condition and once without the second, thus equating the number of trials across conditions. The results were unchanged by these exclusions.

Because the computational models employed here assume that the outcomes of one option are not informative of the outcomes of the other option, in all conditions, outcome probabilities were truly independent across option pairs (although on average anti-correlated in the asymmetric and reversal conditions). Thus, in the symmetric condition, in a given trial, the obtained and forgone outcomes were the same in 50% of trials; in the asymmetric condition this was the case in 37.5% of trials; finally, in the reversal condition this was the case in 28.2% of trials.

### Behavioural analyses

Our main independent variable (IV) of interest was age, which we modelled as a continuous variable. We then assessed the associations between age and five dependent variables (DVs).

#### Choice variables

The three central DVs of interest were choice-related variables:

1) *Accuracy*. In asymmetric and reversal trials, where one option was more advantageous than the other, we focussed on accuracy. Choices in these trials were coded as 1 when participants chose the option with the higher win probability, and 0 otherwise. This variable allows us to address the predictions that age increases accuracy in stationary learning conditions (P1) more than volatile ones (P2).
2) *Preferred choice rate*. In symmetric trials, since options were equally advantageous and accuracy cannot be established, we focussed on the extent to which participants developed a preference for one of the options. We defined the preferred option as the most frequently chosen option, i.e. chosen by the participant on more than 50% of the trials. We then coded choices as 1 when they coincided with the preferred option, and 0 otherwise. This variable allows us to address the prediction that age increases the tendency to choose the same option (choice conservatism) when both options are equally advantageous (P3).
3) *Win-stay/lose-shift*. Across all trials, we focussed on win-stay/lose-shift behaviour. Choices were coded as 1 if participants either chose the same option as in the previous trial after winning in the previous trial, or if they switched to the other option after losing on the previous trial. Choices were coded as 0 otherwise. To distinguish between win-stay and lose-shift, an additional regressor was used to code trials based on whether participants had won or lost on the previous trial. These were coded as a two-level factor with levels “win” vs. “lose”. This variable allows us to assess the prediction that age increases the asymmetry between the tendency to stay after a win and the tendency to shift after a loss (P4).

#### Time variables

As additional DVs of interest, we focussed on two time-related variables:

4) Decision times (DTs). On all trials, we recorded how long it took participants to reach a decision. This is the time between when the symbols were presented and when participants made their choice.
5) Outcome observation times (OOTs). On all trials, we recorded the time spent observing the outcomes of choices. This is the time between when the outcomes were presented and when participants pressed the same key they used to make their choice to pass to the next trial.

Both time measures were log-transformed as this better approximated a normal distribution. These variables allow to assess the secondary hypothesis that age-related changes in reinforcement learning are associated with changes in deliberation times or impulsivity.

To retain all data points whilst accounting for non-independence of observations (e.g., the fact that multiple responses came from the same participant) these 5 DVs were analysed using separate generalized mixed effects models (GLMMs), the details of which are described in the Supplementary Material (Supplementary Material 2). In addition, because age trends vary in their shape, depending on the cognitive processes involved (e.g., Fuhrmann et al., 2015; Laube et al., 2020), we compared different candidate functions of age effects. Out of a linear, quadratic, cubic, logarithmic and inverse functions linking age to reinforcement learning accuracy, AIC suggested that the linear function provided the best fit to the data (see Supplementary Material 2 for details).

#### Non-verbal reasoning

Because non-verbal reasoning abilities develop markedly during adolescence (Chierchia et al., 2019) and have been suggested to predict reinforcement learning performance (Nussenbaum et al., 2021), the matrix reasoning subscale of the WASI (Wechsler, 1999) was also collected after the main task. Since controlling for this variable did not alter our main findings, these are discussed in the Supplementary Material (Supplementary Material 3 for details).

### Data Exclusion

We excluded trials in which the response time was less than 100 ms or larger than 10 s (51 trials out of 7392, less than 1%).

### Computational analyses

Following previous studies (e.g., Palminteri et al., 2015, 2017), we fitted the data with a confirmatory learning model. The model estimates the expected values (Q-values) of each option, that is, the reward participants expect to receive when choosing that option based on its trial-by-trial reward history. The Q-values start at 0, which corresponds to the *a priori* expectation of a 50% chance of winning one point, and a 50% chance of losing one point. After every trial t, the values of the chosen option (QC) and of the unchosen option (QU) are respectively updated according to the following rules:

(1) $$Q_{C}(t+1)=Q_{C}(t)+\begin{array}{c} LR^{Con}PE_{C}(t)\;\;if\;PE_{C}(t)>0\\ LR^{Dis}PE_{C}(t)\;\;if\;PE_{C}(t)<0 \end{array}$$

(2) $$Q_{U}(t+1)=Q_{U}(t)+\begin{array}{c} LR^{Con}PE_{U}(t)\;\;if\;PE_{U}(t)<0\\ LR^{Dis}PE_{U}(t)\;\;if\;PE_{U}(t)>0 \end{array}$$

*PE_C_(t)* and *PE_U_(t)* refer to the prediction errors of the chosen and unchosen options respectively, i.e., how much the actual outcomes differ from the expected outcomes, and can be calculated as:

(3) $$PE_{i}(t)=R_{i}(t)-Q_{i}(t)$$

Where the subscript *i* is C in equation (1) and U in equation (2), *R_i_(t)* is the actual outcome and *Q_i_(t)* is the predicted outcome. The learning rates, *LR^Con^* and *LR^Dis^*, are scaling parameters that adjust the amplitude of value changes from one trial to the next. The confirmatory learning rate *LR^Con^* is used when the chosen outcome is better than predicted (equation 1) or when the unchosen outcome is worse than predicted (equation 2). The disconfirmatory learning rate *LR^Dis^* is used when the chosen outcome is worse than predicted or the unchosen outcome is better than predicted. In other words, *LR^Con^* and *LR^Dis^* quantify sensitivity to confirmatory and disconfirmatory evidence, respectively.

Finally, the probability/likelihood of selecting an option was estimated with a softmax rule:

(4) $$P_{C}(t)=\frac{e^{\beta Q_{C}(t)}}{e^{\beta Q_{C}(t)}+e^{\beta Q_{U}(t)}}$$

This is a standard stochastic decision rule that calculates the probability of selecting one of a set of options according to their associated values. The inverse temperature, β, is another scaling parameter that adjusts the stochasticity of decision making.

We compared four reinforcement learning models. As a baseline model, we used a random model (Wilson & Collins, 2019), which assumes that participants choose randomly but with a potential bias towards one of the two options (which is captured by the single “bias”-related parameter). Because of previously reported differences in the way adolescent and adults learn from counterfactual outcomes, we also fit an “information” model (Palminteri et al., 2016), in which two learning rates are shaped by the outcomes of the chosen and unchosen options, respectively. To control for a positivity bias (Palminteri et al., 2017), we also fit a “valence model”, in which learning two learning rates, *LR^Pos^* and *LR^Neg^* are respectively shaped by positive and negative outcomes, regardless of whether these are chosen or unchosen options. A full model with 4 learning rates (for each possible combination of positive/negative prediction errors vs. chosen/unchosen outcomes) was not included in the model comparison procedure because previous work has shown that it is largely outperformed by the confirmation model (Palminteri et al., 2017). However, we separately fit this full model to address the question of whether adults and adolescents might differ in their use of counterfactual information. This was not the case (see Supplementary Material 4).

#### Parameter optimisation and model comparison

We optimised model parameters by minimising the negative log posterior probability of the model.

(5) LPP=log(P(Data∣Model,Parameters))

This approach was chosen as it considers both the likelihood of the models and the likelihood of the parameter values given their priors, and hence it avoids degenerate parameter estimates, which can happen when only the model likelihood is taken into account.

The optimisation was performed using the optimx function with nlminb algorithm (package optimx). For the Q-learning models, the parameter values were constrained to 0.1 < *β* < 100, and 0 < LR < 1, and initialized at 1 and 0.2 respectively. Parameter priors were based on previous studies and were set to *β* (inverse temperature): gamma distribution (1.2, 5); *LR*: beta distribution (1.1, 1.1). All learning rates *LR* had the same prior. For the random model, the bias parameter was constrained to 0 < bias < 1, initialized at 0.2. There was a uniform prior (0,1) on the bias parameter.

A single set of parameters was used to fit data from all conditions, as a previous studies showed similar parameter estimated across conditions (Chambon et al., 2020; Lefebvre et al., 2017; Palminteri et al., 2017). However, because there is an ongoing discussion (Nussenbaum et al., 2021; Nussenbaum & Hartley, 2019) on whether parameters are flexible, and possibly adaptive to different conditions, the parameters were also estimated separately for each condition. The results of this analysis corroborated the findings of the condition-wide analysis (see Supplementary Material 5 for details).

The models were then compared using Bayesian model selection to obtain exceedance probabilities. This allowed us to estimate the proportion of participants who favoured each model and select the model that was more likely than any other compared model to generate the data of a randomly chosen participant. We also assessed protected exceedance probabilities, which correct exceedance probabilities for the possibility that the observed differences in model evidence are due to chance. We used Aikaike weights for the calculation and forwarded them into the function VB_bms from the package bmsR (Lisi, 2021). Finally, after model comparison, we also assessed parameter and model recoverability (Wilson & Collins, 2019).

## Results

### Behavioural results

#### Choice variables

**Fig. 2:**
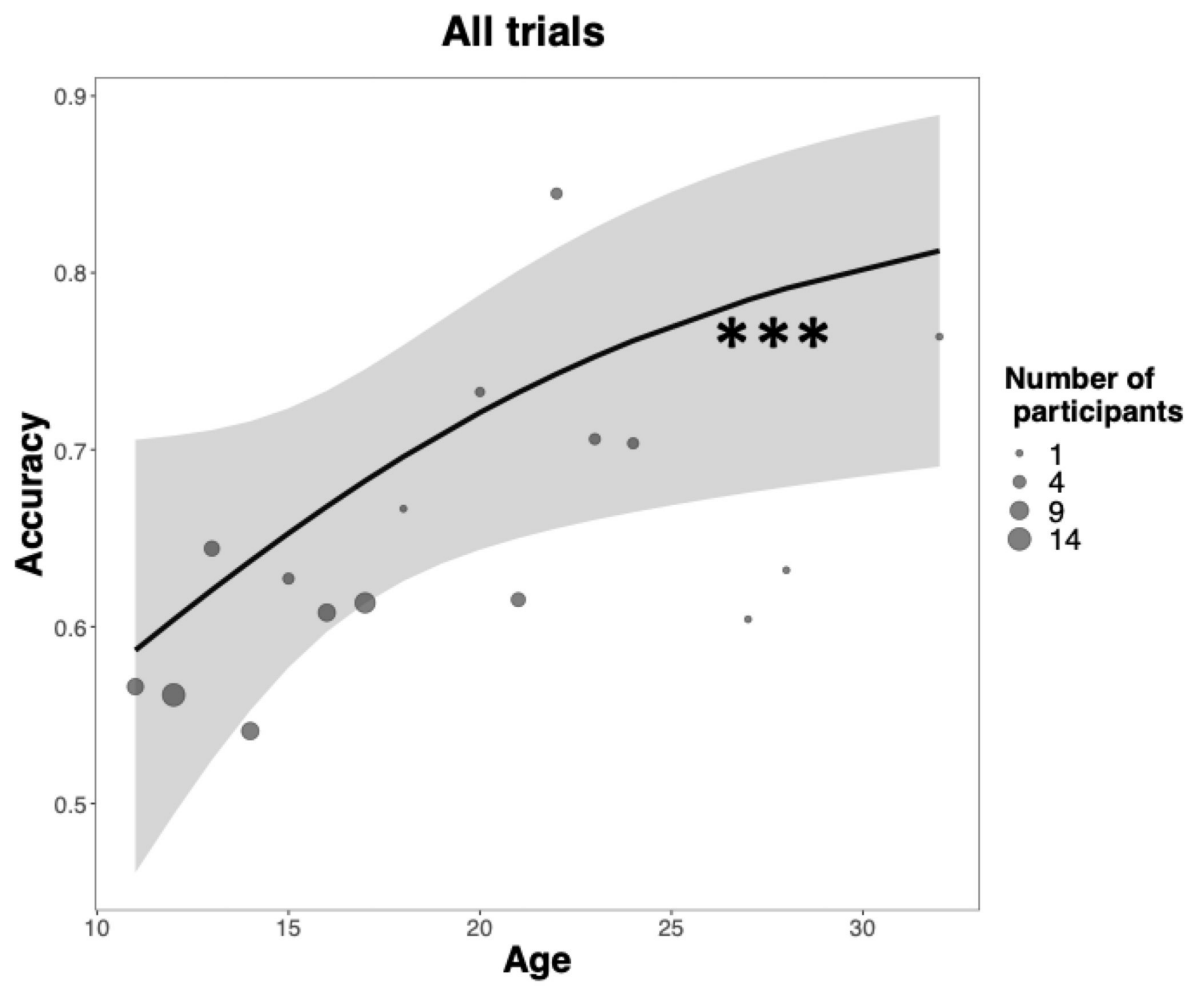
Positive association between age and accuracy across all trials. Solid lines are estimated fixed effects from generalized mixed models with 95% confidence ribbons. Dots are observed grand means, with surface areas proportional to the number of participants. *** p < .001.

The first generalized mixed model (GLMM^Acc^, see Supplementary Material 2) revealed a significant association between age and accuracy (χ^2^(1) = 22.87, p < .001) (Fig. 2): age was associated with a linear increase in the (log) odds of choosing the option with the highest probability of winning (slope = 0.15, SE = .029). These results support prediction P1, that age and accuracy are positively related in stationary and asymmetric learning environments. The model further revealed a significant trend of trials (χ^2^(1) = 57.76, p < .001), which were also associated with an increase in the probability of accurate choices (slope = 0.08, SE = 0.01, p < .001). In addition, age and trials interacted (χ^2^(1) = 25.57, p < .001), such that older participants were more likely to learn as trials progressed, or learned more efficiently, than younger participants. This interaction of trials and age on accuracy was not constant across conditions, as demonstrated by a significant 3-way interaction between age, trial and condition (χ^2^(2) = 20.05, p < .001) (Fig. 7 and Supplementary Material 6): the beneficial impact of age on accuracy was decreased in early post-reversal trials relative to pre-reversal trials, with age decreasing the probability of responding correctly in the first three post-reversal trials (ps <.05). These results support prediction P2, that age-related benefits in accuracy are reduced in more volatile learning environments. Further exploratory comparisons showed that the positive association between trials and accuracy was greater in pre-reversal than asymmetric trials for participants aged 17 or older (ps < .05), but not for younger participants (ps >0.3).

**Fig. 3:**
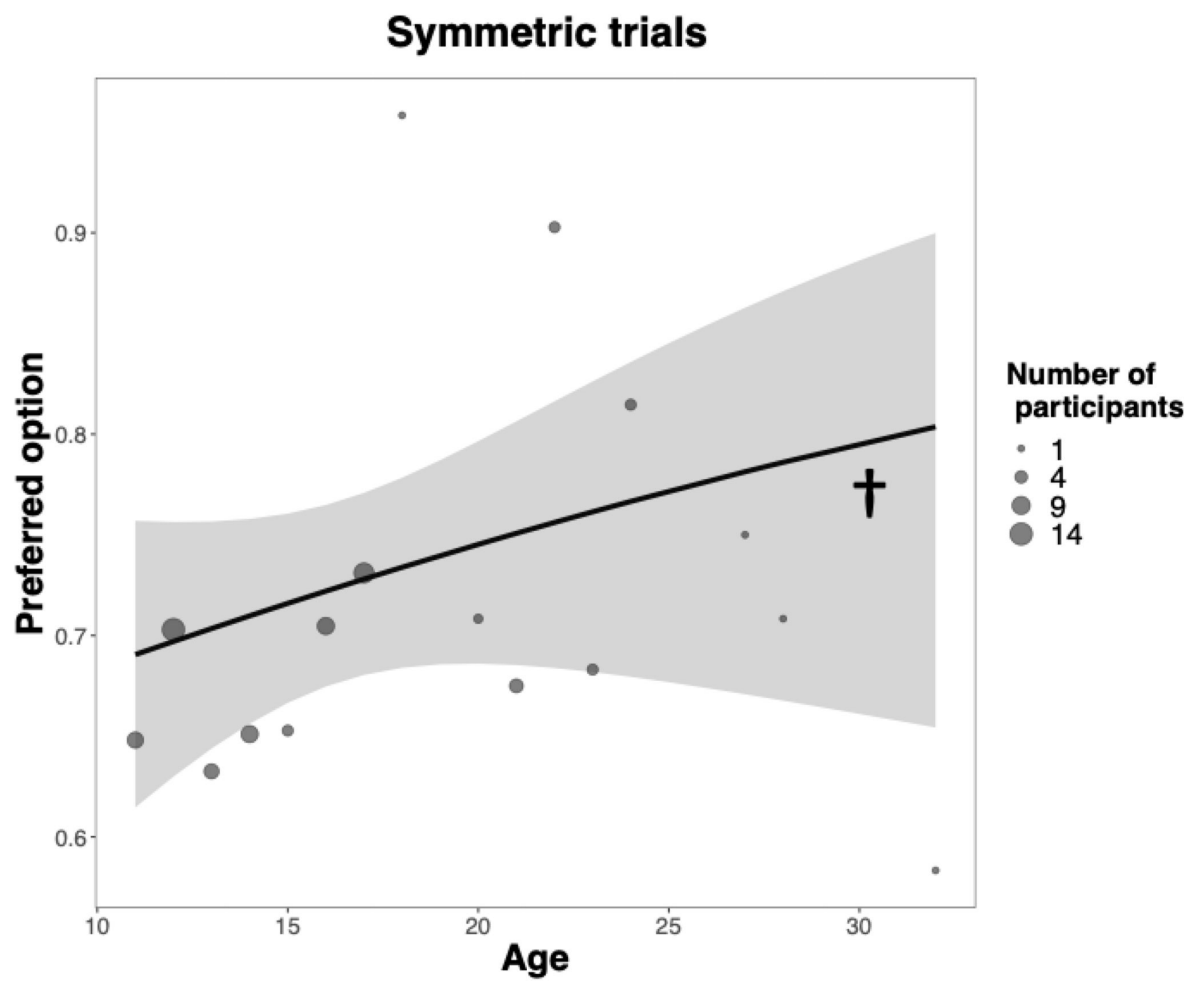
No significant association between age and preferred option choice rate in symmetric trials, in which options are equally advantageous. Solid lines are estimated fixed effects from generalized mixed models with 95% confidence ribbons. Dots are observed grand-means, with surface areas proportional to the number of participants. † p = .090.

A second GLMM on symmetric trials, in which both options are equally advantageous, focussed on whether age modulated choice conservatism, that is, the extent to which participants chose a preferred option (the option chosen on more than 50% of the trials) (GLMM^Pref^, see Supplementary Material 2). This model revealed a positive, but not significant, association between age and conservatism (χ^2^(1) = 2.73, p = .098) (Fig. 3). This result does not support prediction p3 that conservative preferences would increase with age in symmetric learning environments.

**Fig. 4:**
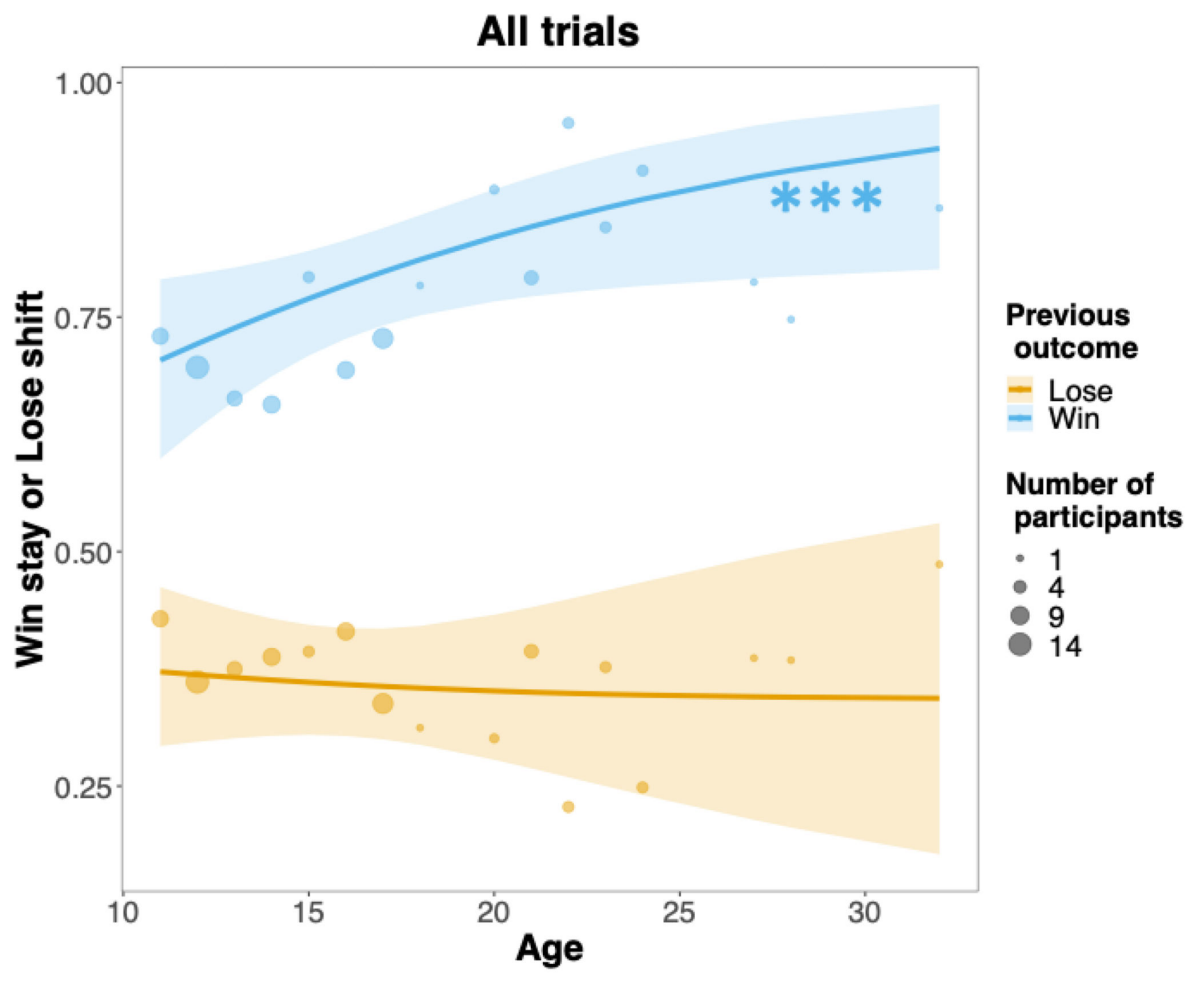
Win-stay/lose-shift asymmetry increased with age across all trials. This was mainly driven by staying after wins (vs. switching after losses). Solid lines are estimated fixed effects from generalized mixed models with 95% confidence ribbons. Dots are observed grand means, with surface areas proportional to the number of participants. *** p < .001.

A third mixed model (GLMM^WSLS^, see Supplementary Material 2) revealed a significant main effect of the previous outcome on win-stay/lose-shift behaviour (χ^2^(1) = 141.88, p < .001). Specifically, participants were more likely to stay after a win than to switch after a loss (contrast_stay/win – switch/loss_ = 1.93, SE = 0.162, p < .001). This further interacted with age (χ^2^(1) = 7.31, p = .007). Specifically, increasing age was associated with a linear increase in the log odds that participants would stay after a win (slope = 0.09, SE =0.02, p < .001). However, age did not modulate the extent to which people switched after a loss (slope = -0.008, SE = 0.02, p = .678) and the contrast between these two slopes was significant (slope_stay/win – switch/loss_ = 0.1, SE = 0.04, p = .007). This supports our prediction that WSLS asymmetry would increase with age (Fig. 4). Notably, exploratory post-hoc analyses showed that the age-related increase in WSLS asymmetry was also robust in symmetric trials. In these trials, age was associated with increased log odds of staying after a win (slope = 0.07, SE = 0.02, p < .001) and decreases the log odds of shifting after a loss (slope = -0.05, SE = 0.02, p < .001). This demonstrates that age-related increases in WSLS asymmetry are not a mere by-product of increased learning, because symmetric trials are learning-neutral (see Supplementary Material 7 for further information).

#### Decision time and observation time variables

**Fig. 5:**
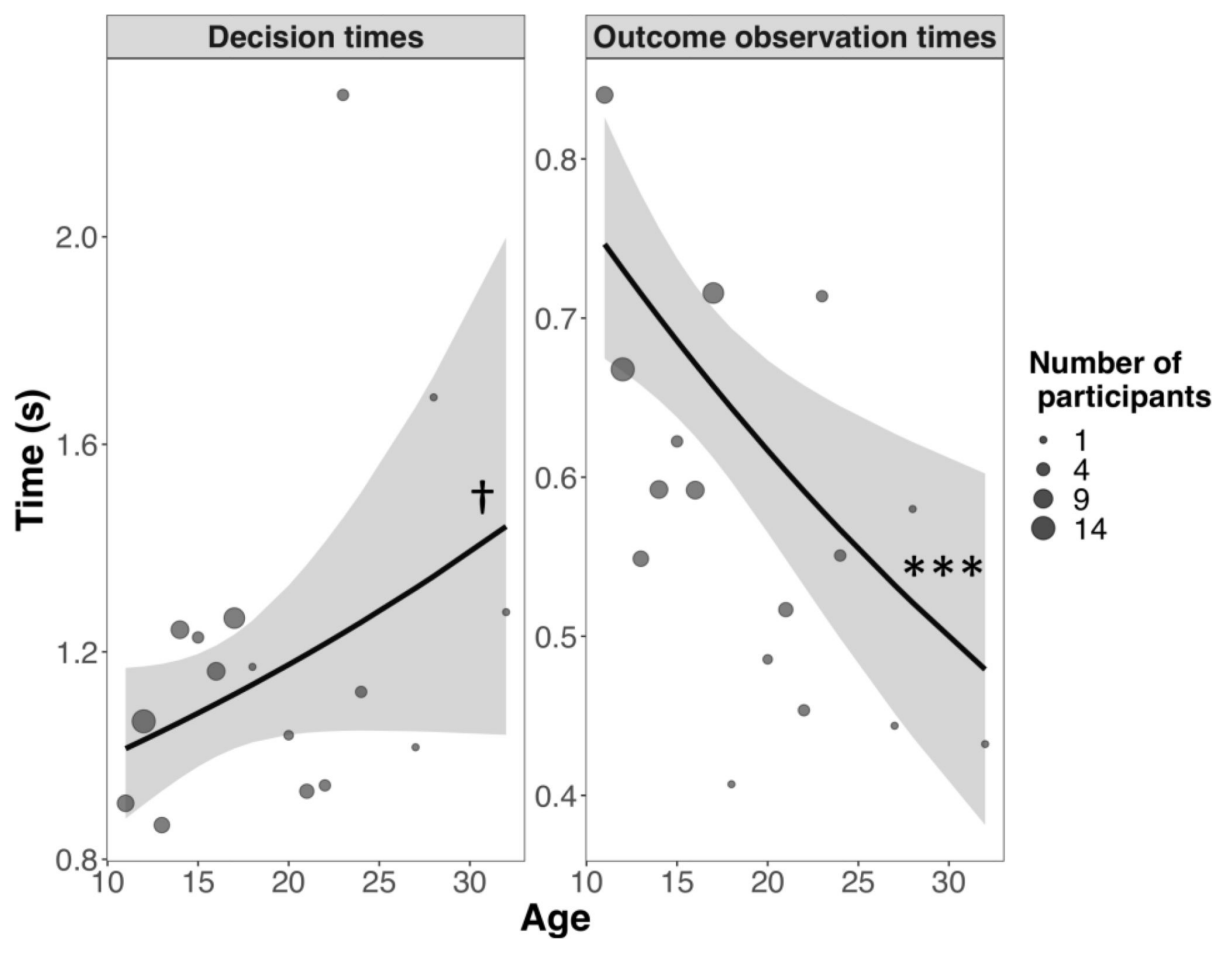
Age, decision times and outcome observation times. No significant association between age and decision times (left panel). Negative association between age and outcome observation times (right panel). Solid lines are estimated fixed effects from generalized mixed models with 95% confidence ribbons. Circles are observed grand medians (for condition-level plots see Supplementary Material 11). *** p < .001, † p <.10.

A first mixed model (GLMM^DT^, see Supplementary Material 2) suggested there was no overall effect of age on decision times (χ^2^(1) = 2.95, p = .086) (Fig. 5, left panel). This was further qualified by an interaction between age and condition (χ^2^(3) = 12.87, p = .005): there was a significant positive association between age and decision times in symmetric trials (slope = 0.03, SE = 0.01, p = .004) but not in the remaining conditions (all ps > .180). The only significant contrasts between these associations (i.e., between the condition-level slopes relating age to decision times) were the contrasts between symmetric and asymmetric trials, and between symmetric and post-reversal trials (both ps_Bonf_ < .05). The contrast between symmetric and pre-reversal trials did not survive Bonferroni correction (ps_Bonf_ = .062). Overall, this model suggested that decision times only increased with age in symmetric trials (see Supplementary Material 11).

A second mixed model (GLMM^OOT^, see Supplementary Material 2) revealed a significant association between age and outcome observation times (χ^2^(1) = 11.93, p < .001): outcome observation times decreased linearly with age (slope = -0.02, SE = 0.006, p < .001) (Fig. 5, right panel). This overall trend was modulated by an interaction with condition (χ^2^(3) = 8.91, p = .031), but no contrasts survived correction for multiple comparisons (all ps_Bonf_ > .069). The same model also revealed a main effect of the current outcome (χ^2^(1) = 171.85, p < .001): outcome observation times were shorter after winning than after losing (contrast_won – lost_ = 0.29, SE 0.02, p < .001). However, this did not interact with age (χ^2^(1) = 0.23, p = 0.637). Follow up exploratory models (GLMM^Acc-DT^ and GLMM^Acc-OOT^, see Supplementary Material 8) revealed significant interactions between age and time-related variables on accuracy (decision times: χ^2^(1) = 4.35, p = .037; outcome observation times: χ^2^(1) = 23.56, p < .001). Longer decision and outcome observation times were associated with decreased accuracy and this negative association increased linearly with age (Supplementary Material 8). The first of these interactions (between age and decision times on accuracy) was no longer significant after removing the 4 men from the sample (χ^2^(1) = 1.97, p = .161), but it remained significant for outcome observation times (χ^2^(1) = 17.26, p < .001).

### Computational results

Model comparisons suggested that the confirmation model, that is, a model with separate confirmatory and disconfirmatory learning rates, best explained the observed behaviour (Table 1). The models displayed adequate parameter and model recoverability (see Supplementary Material 9 for details).

**Fig. 6:**
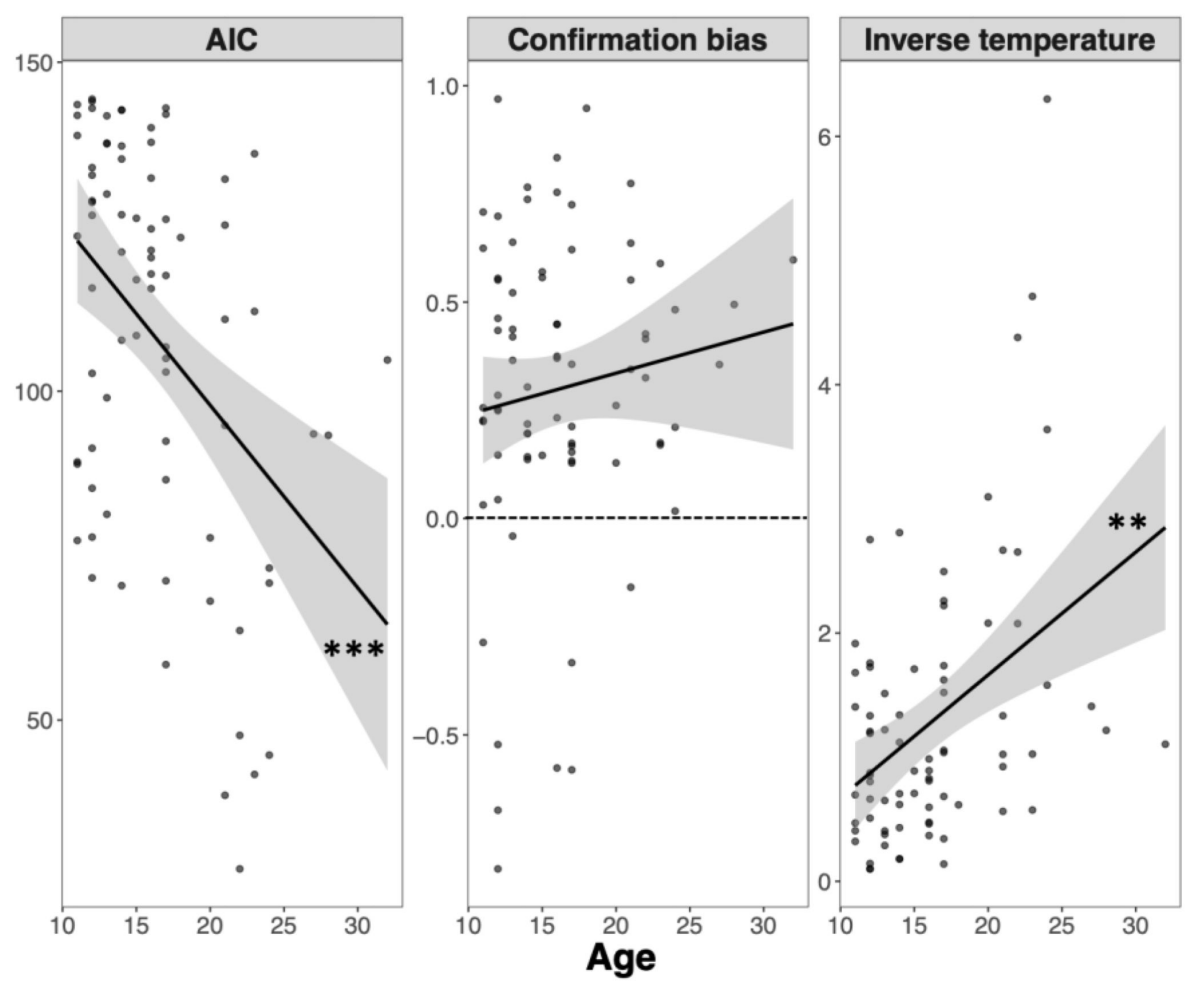
Model fit (AIC) and parameter estimates of the confirmation model as a function of age. The dashed line in the middle panel indicates unbiased values. Asterisks indicate p-values of Spearman’s rank correlations. ***p < .001, **p < .01.

Rank correlations (Spearman) further suggested that the fit of the confirmation model improved with age (ρ = 0.4, p < .001) (Fig. 6, left panel), particularly relative to the random model (see Supplementary Material 10 for further details). In terms of model parameters, the confirmation model showed that, in both adults and adolescents, the mean difference between confirmatory and disconfirmatory learning rates (*LR^Con^* – *LR^Dis^*) was positive (*M*_Adults_ = 0.39, *SD* = 0.26; *M_Adolescents_* = 0.27, *SD* = 0.38), and significantly different from 0 (Wilcoxon sign-rank tests, ps < .001). This suggests that both age groups displayed choice confirmation bias. However, the magnitude of the bias was not associated with age (ρ = 0.059, p < .614) (Fig. 6, middle panel). In contrast, inverse temperature showed a positive association with age (ρ = 0.4, p = 0.001) (Fig. 6, right panel). Overall, these findings suggest that, between adolescence and adulthood, increasing age is associated with an increased likelihood of adopting confirmatory learning strategies, coupled with lower levels of noise or exploration in confirmatory learning, and no difference in the magnitude of choice confirmation bias.

**Fig. 7:**
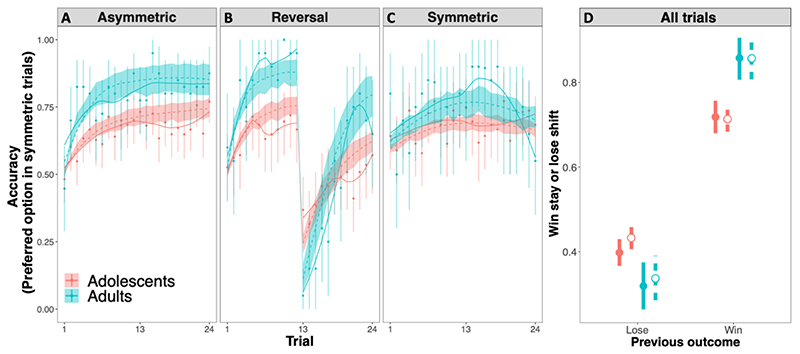
Observed and simulated data. Full lines and points are observed data, dashed lines and empty points are simulations. These simulations tracked each of the four confirmatory learning patterns described above. Panel A. Adults were more accurate than adolescents in asymmetric trials. Panel B. Age-related accuracy advantages were reduced in post-reversal trials. Panel C. Adults and adolescents displayed a similar tendency to repeatedly select the same option in symmetric trials. Panel D. Across trials, adults were more likely than adolescents to stay on an option that delivered a reward on the preceding trial, than to shift away from an option that just delivered a punishment. Error bars and confidence ribbons are bootstrapped 95% confidence intervals.

Based on these estimated participant-level parameters we simulated participants’ behaviour for each of the four predicted behavioural patterns of confirmatory learning. The fit between the simulated and observed data appeared reasonable (Fig. 7), in that they recovered the age-related changes illustrated in the behavioural analyses: 1) heightened accuracy in asymmetric and pre-reversal trials (panel A and left-side of panel B), 2) decreased accuracy in post-reversal trials (right side of panel B), 3) increased selection of preferred options in symmetric trials (panel C), and 4) increased win-stay/lose-shift asymmetry across trials (panel D).

**Fig. 8:**
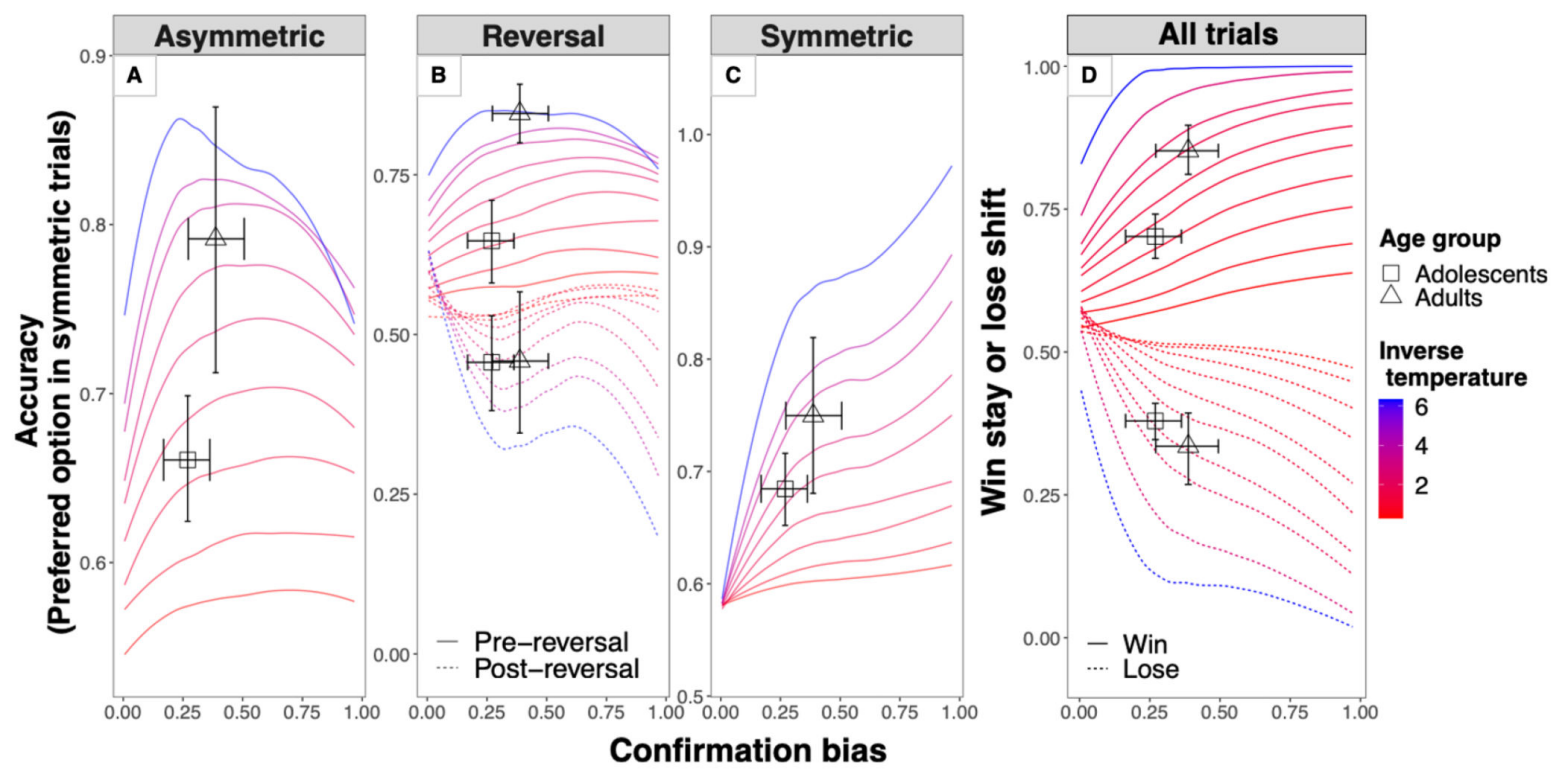
Behavioural patterns of confirmatory learning as a function of confirmation bias and inverse temperature. Lines are simulated behaviours across a range of parameter values of confirmation bias (*LR^Con^* – *LR^Dis^*, on the x-axis) and inverse temperature (colour gradient). These simulations suggest that, frequently, confirmation bias and inverse temperature both predict increased accuracy in asymmetric trials (A), decreased accuracy in post-reversal trials (dashed lines panel B), increased selection of preferred options in symmetric trials (C) and increased discrepancy between win/stay vs. lose/shift behaviour (respectively, solid vs. dashed lines of panel D).

Finally, to further qualify how learnings rates and inverse temperature contributed to these behavioural patterns, we simulated those behaviours for a range of possible parameter values (i.e., all possible combinations of each decile of each parameter) and assessed how the observed values fall within this this space. Fig. 8 suggests that confirmation bias and inverse temperature can frequently compete to explain variance in each of the predicted behavioural patterns, and that age-related differences in these patterns are more likely to be captured by inverse temperature than confirmation bias.

In line with this notion of frequent trade-offs between confirmation bias and inverse temperature, both estimated parameters showed similar directional associations with a subset of the predicted behavioural patterns, though inverse temperature explained substantially more variance (Table 2).

## Discussion

This study aimed to assess whether confirmatory reinforcement learning, the tendency to learn more from confirmatory than from disconfirmatory reinforcers, changes with age between adolescence and early adulthood. In line with our predictions, in a standard reinforcement learning task, we found that performance improved with age between 11 and 32 years in a stationary condition (prediction P1), but that these age-related improvements were reduced in a reversal learning condition in which a previously advantageous option suddenly became disadvantageous (P2). Age did not affect participants’ tendency to repeatedly select the same option when both options were equally advantageous (in contrast to P3). Participants of all ages were more likely to repeat choices that had just been rewarded more than they were to switch away from choices that had just been punished, but the magnitude of this win-stay/lose-shift asymmetry increased with age (P4). At the computational level, a confirmation model, which allows confirmatory and disconfirmatory learning rates to vary separately, provided a better fit to the data than alternative models (P5), and the fit improved with age between adolescence and adulthood. The model revealed that age-related differences in confirmatory learning were best explained by differences in inverse temperature, i.e., noise or exploration, rather than the magnitude of the confirmation bias itself. Finally, we found that outcome observation times were greater for younger participants than for older participants, suggesting that age-related improvements in reinforcement learning are unlikely to be explained by developmental trajectories of impulsivity (Steinberg, 2010) or reduced attention to decision outcomes.

Our results suggest that learning becomes increasingly confirmatory between adolescence and adulthood. While this can be beneficial when reinforcement contingencies are asymmetric and stationary (Lefebvre et al., 2022) (P1), it could lead to momentary disadvantages when environments change unexpectedly (P2). It should be noted that age-related effects in pre- vs. post-reversal accuracy are not entirely separable. It is plausible that, because older participants learned faster in pre-reversal trials, they also encountered larger disadvantages when learning contingencies changed. In line with this, the adult disadvantage in post-reversal trials was only temporary (i.e., it was only observed in early post-reversal trials). Indeed, while older participants incurred larger accuracy costs than younger participants when contingencies reversed (i.e., they “crashed harder”), they also recovered faster, and continued to outperform younger participants in later post-reversal trials (e.g., Fig. 7, panel B). Despite this, though not originally predicted, the finding that the adult advantage in accuracy was amplified in pre-reversal, relative to asymmetric trials, further supports the view that age-related benefits in reinforcement learning could be related to option asymmetries, that is, how differentiable options are in terms of reward/punishment probabilities. Indeed, what we labelled as pre-reversal and asymmetric trials were both asymmetric trials that differed in the magnitude of the asymmetry. Specifically, in pre-reversal trials one option was associated with an 83% probability of a reward and the other option with 17%. In contrast, in asymmetric trials, the reward probabilities were 75% and 25%. Consequently, the options were more differentiable (i.e., more asymmetric) in one condition than in the other. We speculate that this increased asymmetry might have led to the amplification of age-related learning advantages.

The results above shed light on the type of environments that might amplify or reduce age-related advantages in learning. However, several of our results also show how age-related changes in learning are not limited to material advantages or disadvantages associated with cumulative reward/punishment, but differences in learning style more broadly. For example, across each of the environments tested, age was associated with an increased tendency to stay after wins (P4), and no change (or even a decrease) in the tendency to switch after a loss. Importantly, this age-related increase in win-stay/lose-shift asymmetry was also observed in symmetric trials, which are performance neutral. This suggests that the age-related increase in win-stay/lose-shift asymmetry is not merely a by-product of better learning with age. Rather, we speculate that, in symmetric trials, adults behaved as if their choices had been confirmed, even though both options had the same objective chances of being rewarded.

Similarly, we had also predicted (P3) that adults might be more conservative in their choices than adolescents (in symmetric trials), and that is, that they would tend to stay with one option more than the other when both are equally advantageous. This prediction was not supported by our data, yet we interpret this null finding with caution as we observed a trend in the hypothesized direction. If the association between age and choice conservatism in symmetric trials is small, a more highly powered study might be able to detect it. We also note that age was not associated with decision times in any condition except for the symmetric condition, where age predicted longer decision times. This suggests that attempting to maximize efficiency in environments where efficiency cannot be attained, such as in symmetric environments, might be associated with cognitive costs, such as deliberation times, rather than material costs.

At the computational level, we found that, between adolescence and early adulthood, age-related differences in RL were captured by two features: first, participants became increasingly likely to learn more from confirmatory and disconfirmatory outcomes. Second, RL became less noisy. The first result is supported by observation that the confirmation model fits improved with age, the second by the observation that inverse temperature declined with age. Indeed, while inverse temperature can sometimes be interpreted as noise or exploration, the interpretation of this parameter in our paradigm is more likely related to noise (also called random exploration or selection noise) than to exploration (sometimes called strategic exploration) (Findling et al., 2019; Nussenbaum & Hartley, 2019). In fact, because our paradigm provided counterfactual feedback (i.e., participants were also shown the outcomes of the options they did not choose), exploration/exploitation trade-offs were minimized.

Age-related decreases in inverse temperature during adolescence have been observed many times before (Nussenbaum & Hartley, 2019 for a review). This is also consistent with the recently advanced notion that, much like statistical learning algorithms, which cool off as they approach optimal solutions, learners become less stochastic and more accurate with age (Giron et al., 2022; Gopnik, 2020). However, we speculate that not all noise/inverse temperatures are equal, because this might also depend on the models they are embedded in. One of the proposed functions of confirmatory learning is that, by emphasizing confirmatory outcomes, this learning style can artificially decrease psychological noise (e.g., inverse temperature) to buffer the impact of environmental noise (Lefebvre et al., 2022; Qiu et al., 2020). In line with this, our simulations suggest that inverse temperature and confirmation bias (i.e., the magnitude of learning rate asymmetry) frequently compete to explain the behavioural patterns tested here (Fig. 8). Further, beyond simulations, this trade off also partly held true in our data, in that the estimated levels of inverse temperature and confirmation bias made similar directional predictions for a subset of the behavioural patterns (Table 2). However, inverse temperature clearly contributed to a larger extent. Similarly, age differences in RL were captured by differences in inverse temperature but not by the magnitude of the confirmation bias. In summary, we speculate that people do not simply become less noisy with age during adolescence, but they might become less noisy in confirmatory learning.

Finally, one limitation of this study is that it might have been underpowered (see Supplementary Material 1), especially with regards to the older participants. Better powered studies could help address possibly smaller age-related effects that were not detected here (such as choice conservatism in symmetric trials). A second limitation is that our sample was almost entirely composed on women/girls. Because some previous studies have observed gender differences in reinforcement learning (Evans & Hampson, 2015), we recommend caution in generalizing the reported results to men/boys. Finally, cross-sectional studies such as ours can conflate age-related and inter-individual differences. Further longitudinal approaches to similar developmental questions are thus warranted.

## Conclusion

How learning styles change during adolescence is a central question in developmental science. Here, we adopted a computational approach to address how reinforcement learning, one of the building blocks of learning, changes between adolescence and adulthood. At both the behavioural and computational levels, our results suggest that reinforcement learning becomes increasingly confirmatory during adolescence. We found that, during this period of life, young people become more accurate learners in stable learning environment (in which confirmatory evidence is important), but not necessarily in more volatile environments (in which disconfirmatory evidence is important). Between adolescence and early adulthood, participants also became more likely to stay with choices that were recently confirmed, rather than shift away from choices that were disconfirmed. In environments in which learning cannot be improved, adults might also incur higher cognitive costs, such as longer deliberation times. Computationally, age-related differences in reinforcement learning are associated with reduced noise in learning separately about confirmatory and disconfirmatory evidence. These results provide new insights into how learning might change with age during development and could help better tailor learning environments to people of different ages.

## Supplementary Material

Supplementary Figure 1

Supplementary Figure 2

Supplementary Figure 3

Supplementary Figure 4

Supplementary Figure 5

Supplementary Figure 6

Supplementary Figure 7

Supplementary Figure 8

Supplementary Figure 9

Supplementary Figure 10

Supplementary Figure 11

Supplementary Figure 12

Supplementary Information

## Figures and Tables

**Table 1:** Model frequencies and exceedance probabilities

Model	Model frequencies	Exceedance probability	Protected exceedance probability
Confirmation model	0.7	1	1
Information model	0.01	0	0
Valence model	0.03	0	0
Random model	0.26	0	0

**Table 2:** Parameter estimates and confirmatory learning patterns. Rank correlations (Spearman) between behavioural measures of confirmatory learning and two confirmation model parameter estimates: inverse temperature and confirmation bias. Confirmation bias is the difference between learning rates and is either raw: *LR^Con^* – *LR^Dis^*; or normalised: (*LR^Con^* – *LR^Dis^*) / (*LR^Con^* + *LR^Dis^*). ***p < .001, **p<0.01, *p<0.05, °p<0.1, FDR corrected (Benjamini-Hochberg method).

Behavioural pattern	Inverse temperature	Confirmation bias	
		Raw	Normalised
Accuracy (Asymmetric and pre-reversal trials)	0.89***	0.06	0.05
Accuracy (Post-reversal trials)	-0.27*	0	-0.31*
Conservatism (Symmetric trials)	0.41***	0.3*	0.38**
Win-stay (All trials)	0.87***	0.21°	0.11
Lose-shift (All trials)	-0.79***	-0.26*	-0.34**

## Data Availability

We cannot share the data for ethical reasons, because we did not ask participants for permission to make their data publicly available.

## Research highlights

- Reinforcement learning shows age-related improvement during adolescence, but more in stable learning environments compared with volatile learning environments.
- People tend to stay with winning options more than they shift away from losing ones, and this asymmetry increases with age during adolescence.
- Computationally, these changes are captured by a developing confirmatory learning style, in which people learn more from outcomes that confirm rather than disconfirm their choices.
- Age-related differences in confirmatory learning are explained by decreases in stochasticity, rather than changes in the magnitude of the confirmation bias.
