# Supplementary figures and images for: Confirmatory reinforcement learning changes with age during adolescence
Source: Dev Sci. Author manuscript; Available in PMC 2023 Nov 6. (PMC7615280; doi:10.1111/desc.13330)

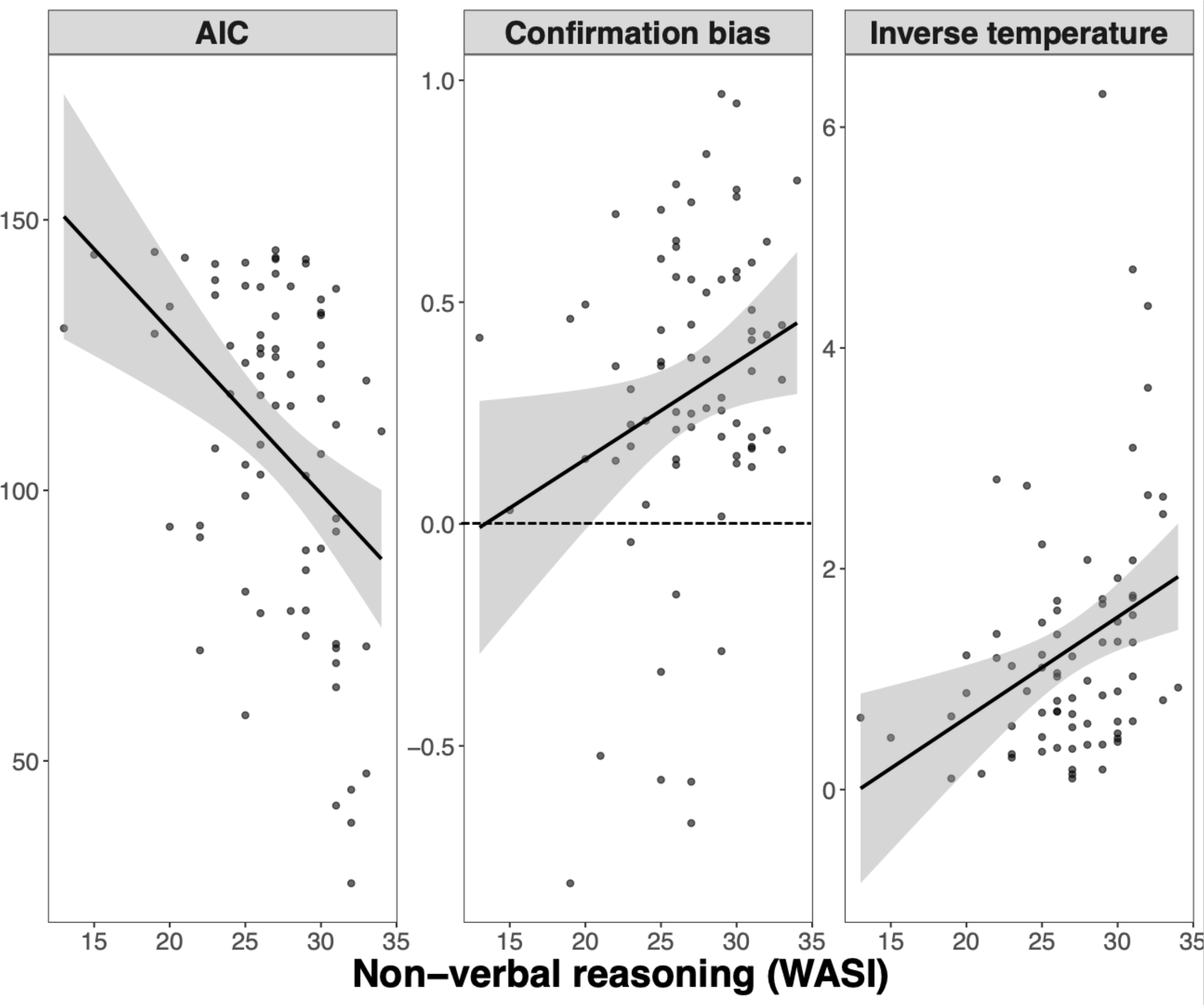

Supplement: Supplementary Figure 1 [file EMS190284-supplement-Supplementary_Figure_1.tiff]

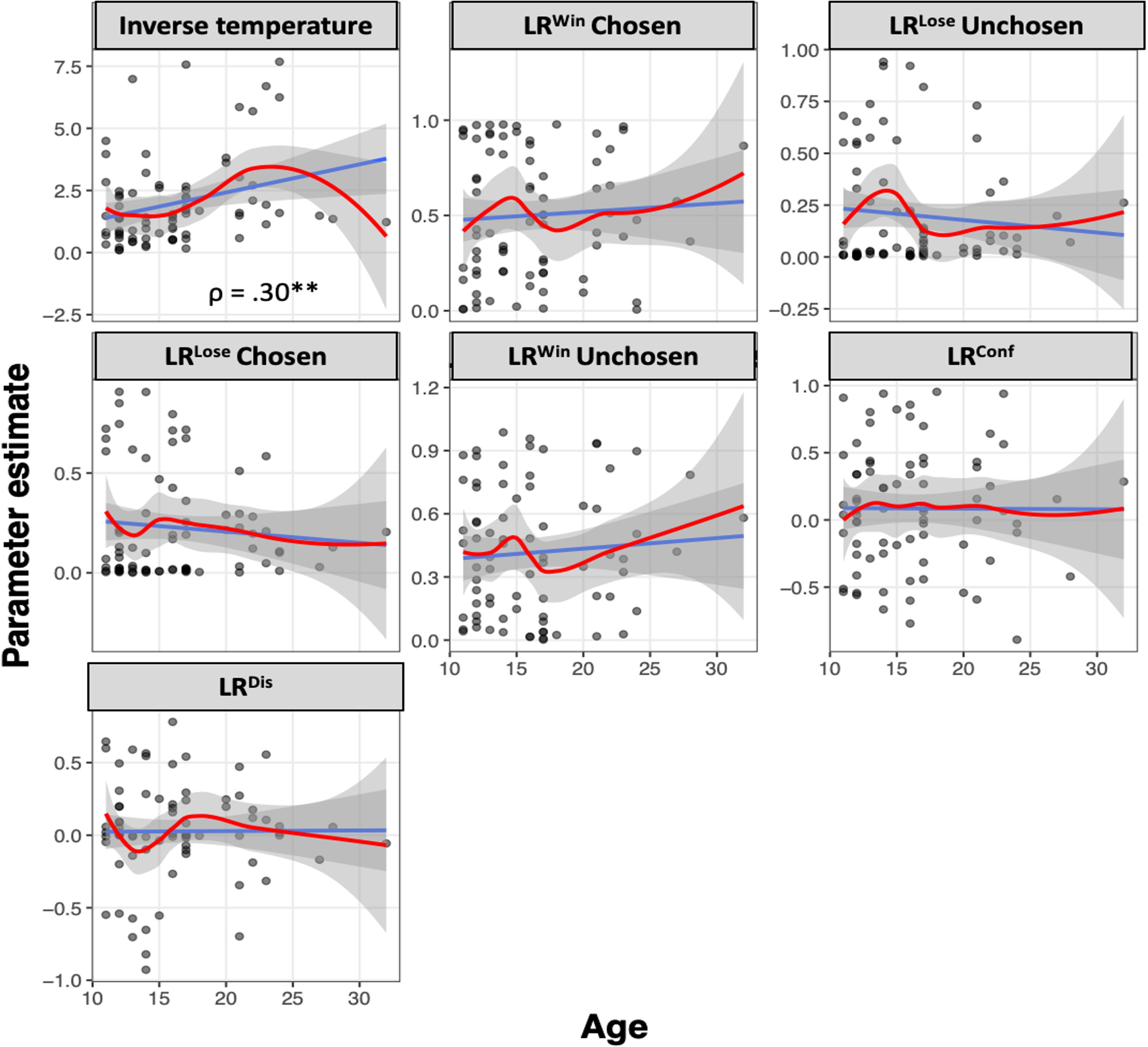

Supplement: Supplementary Figure 2 [file EMS190284-supplement-Supplementary_Figure_2.tiff]

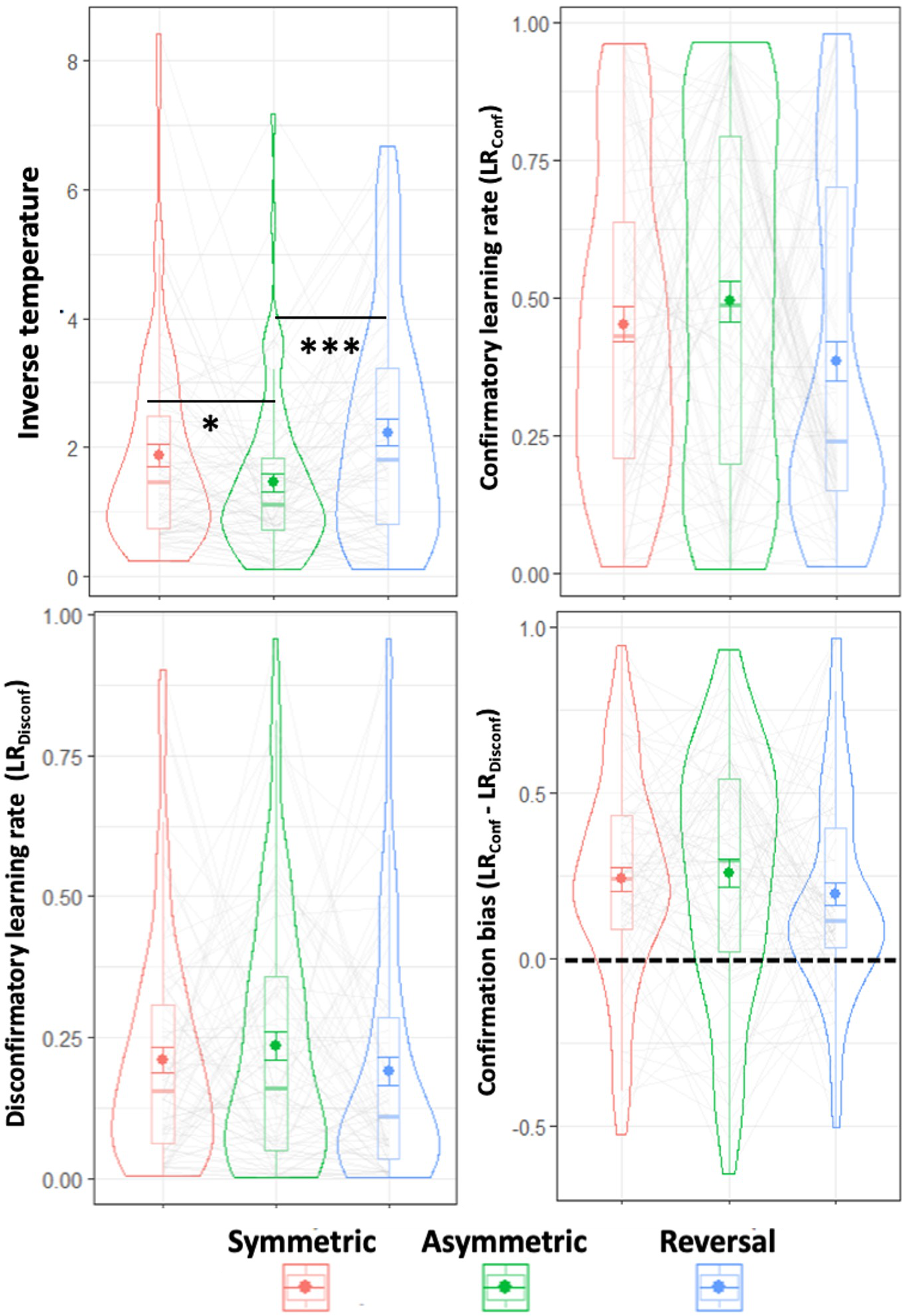

Supplement: Supplementary Figure 3 [file EMS190284-supplement-Supplementary_Figure_3.tiff]

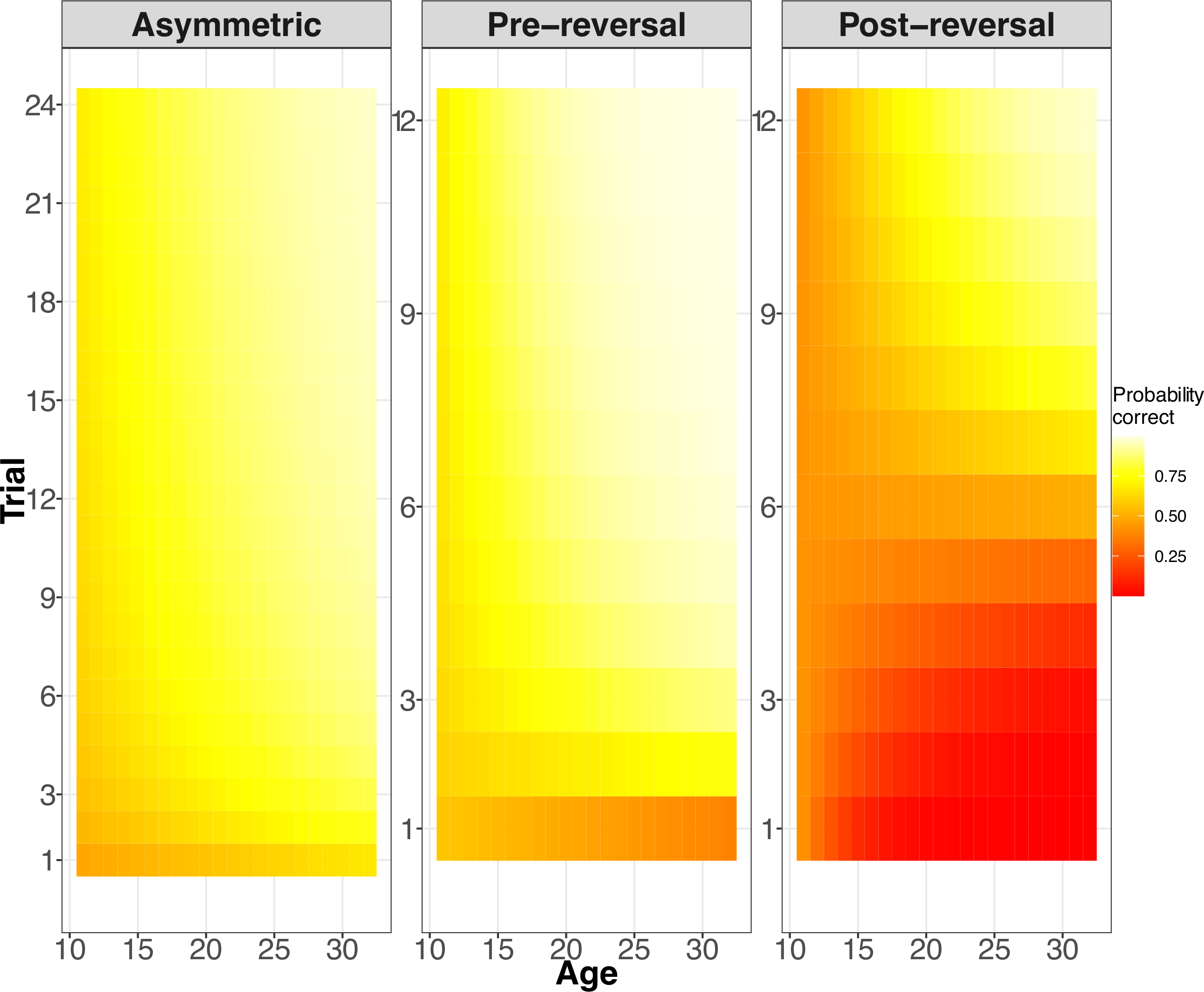

Supplement: Supplementary Figure 4 [file EMS190284-supplement-Supplementary_Figure_4.tiff]

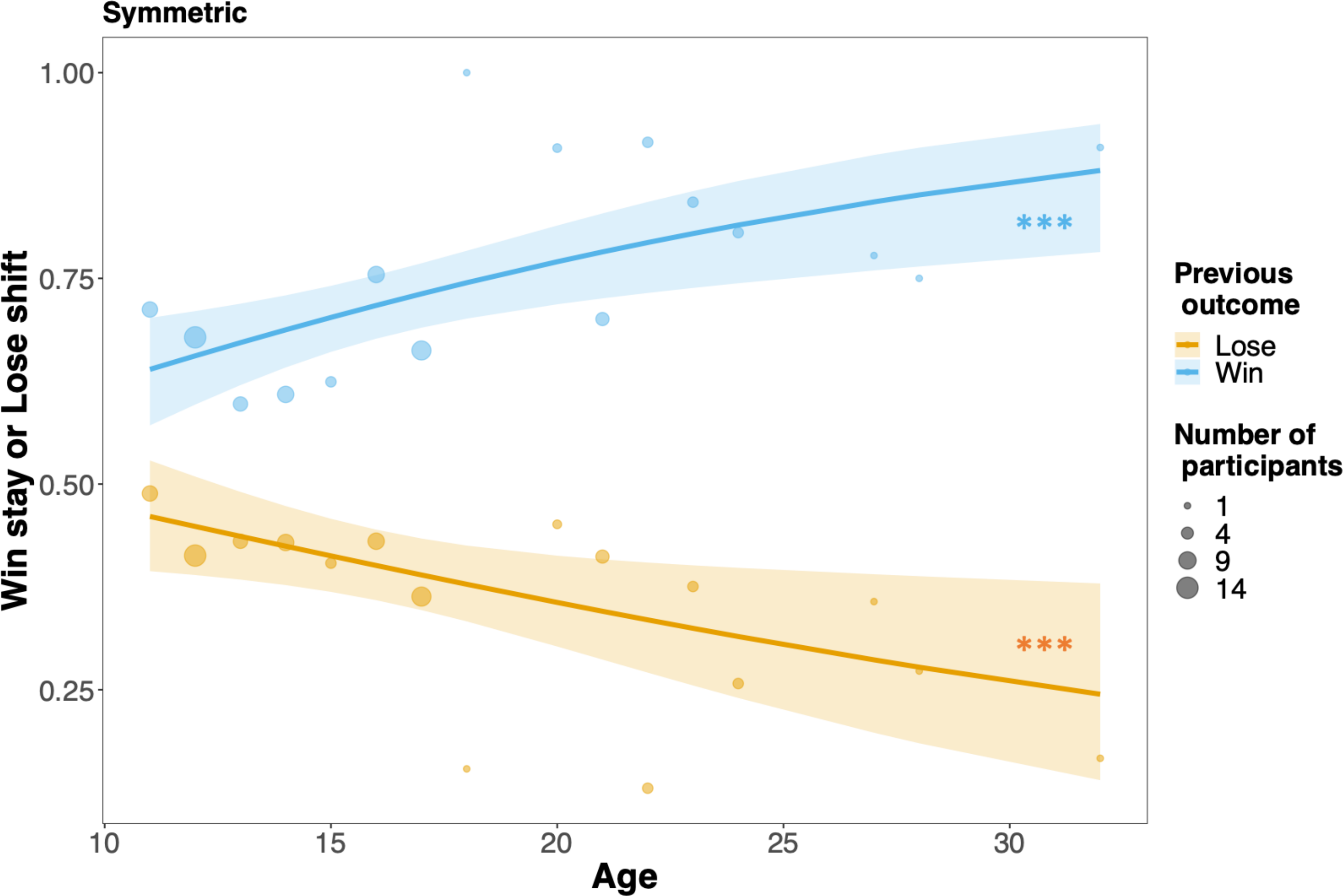

Supplement: Supplementary Figure 5 [file EMS190284-supplement-Supplementary_Figure_5.tiff]

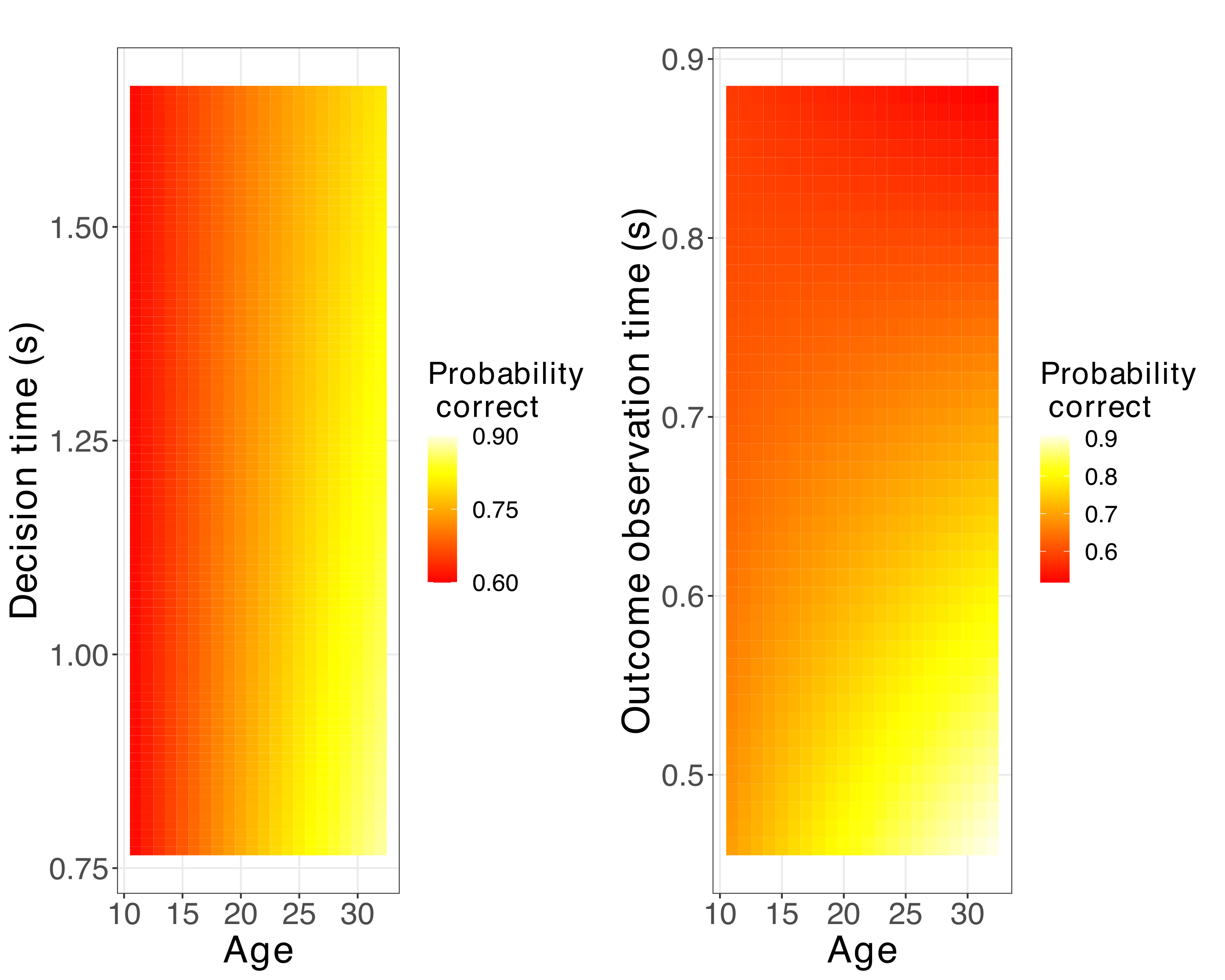

Supplement: Supplementary Figure 6 [file EMS190284-supplement-Supplementary_Figure_6.tiff]

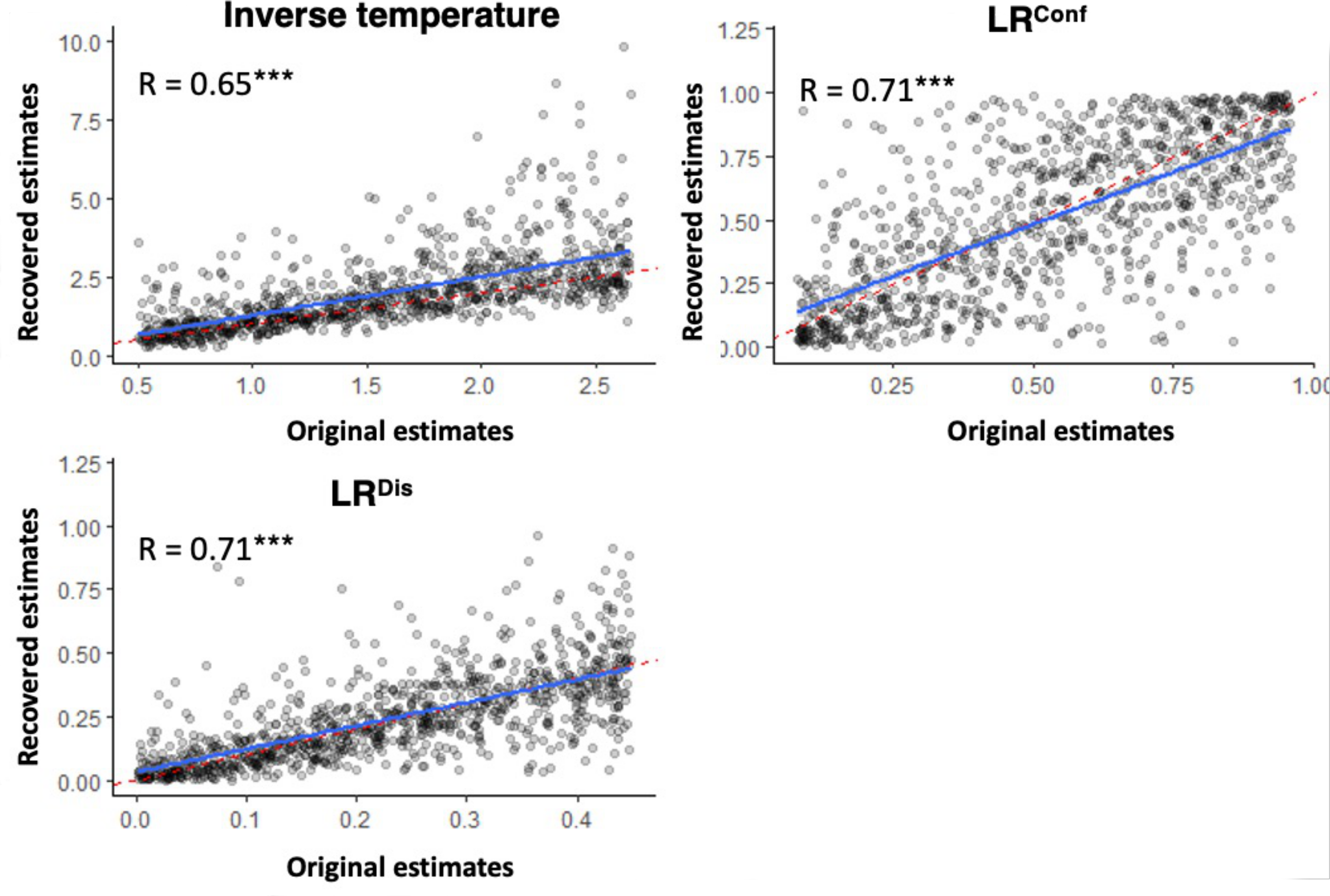

Supplement: Supplementary Figure 7 [file EMS190284-supplement-Supplementary_Figure_7.tiff]

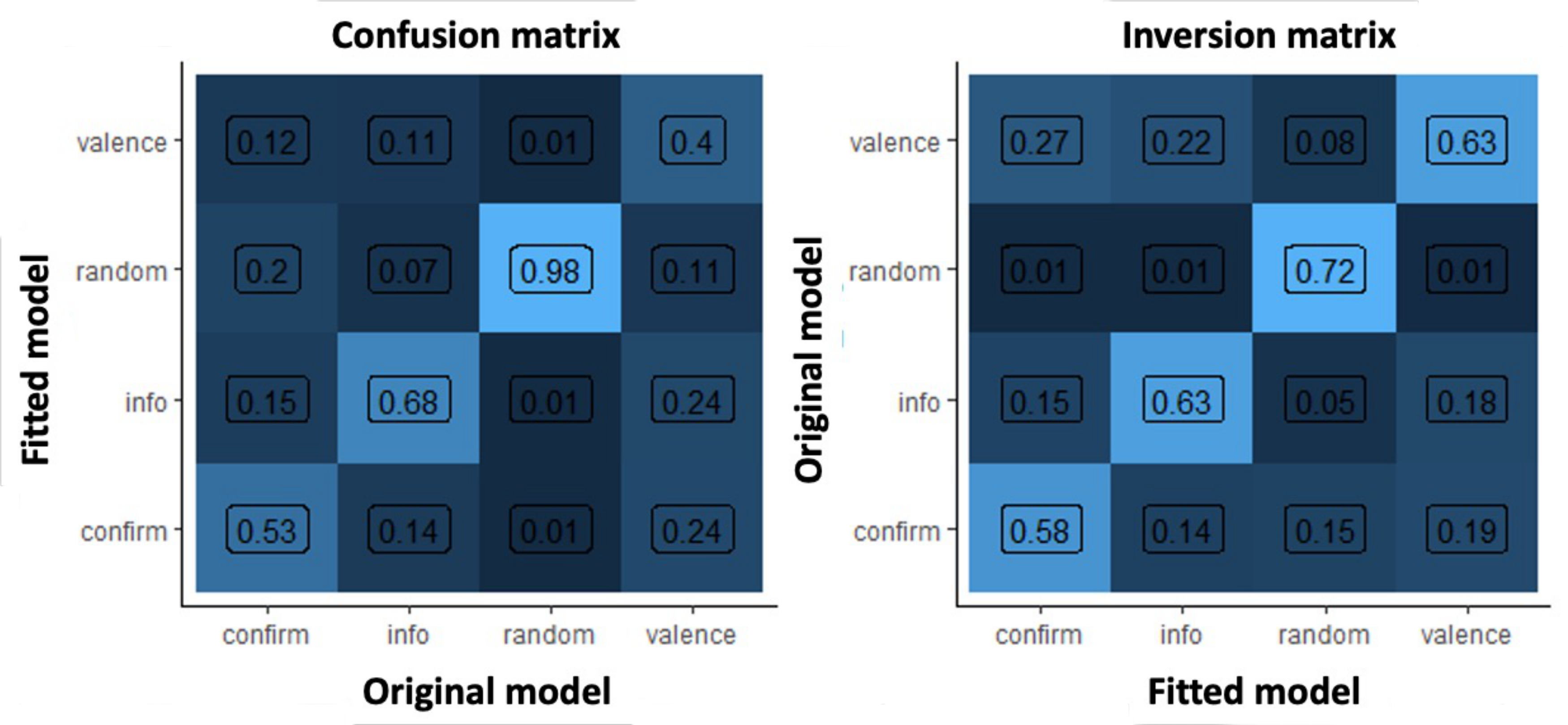

Supplement: Supplementary Figure 8 [file EMS190284-supplement-Supplementary_Figure_8.tiff]

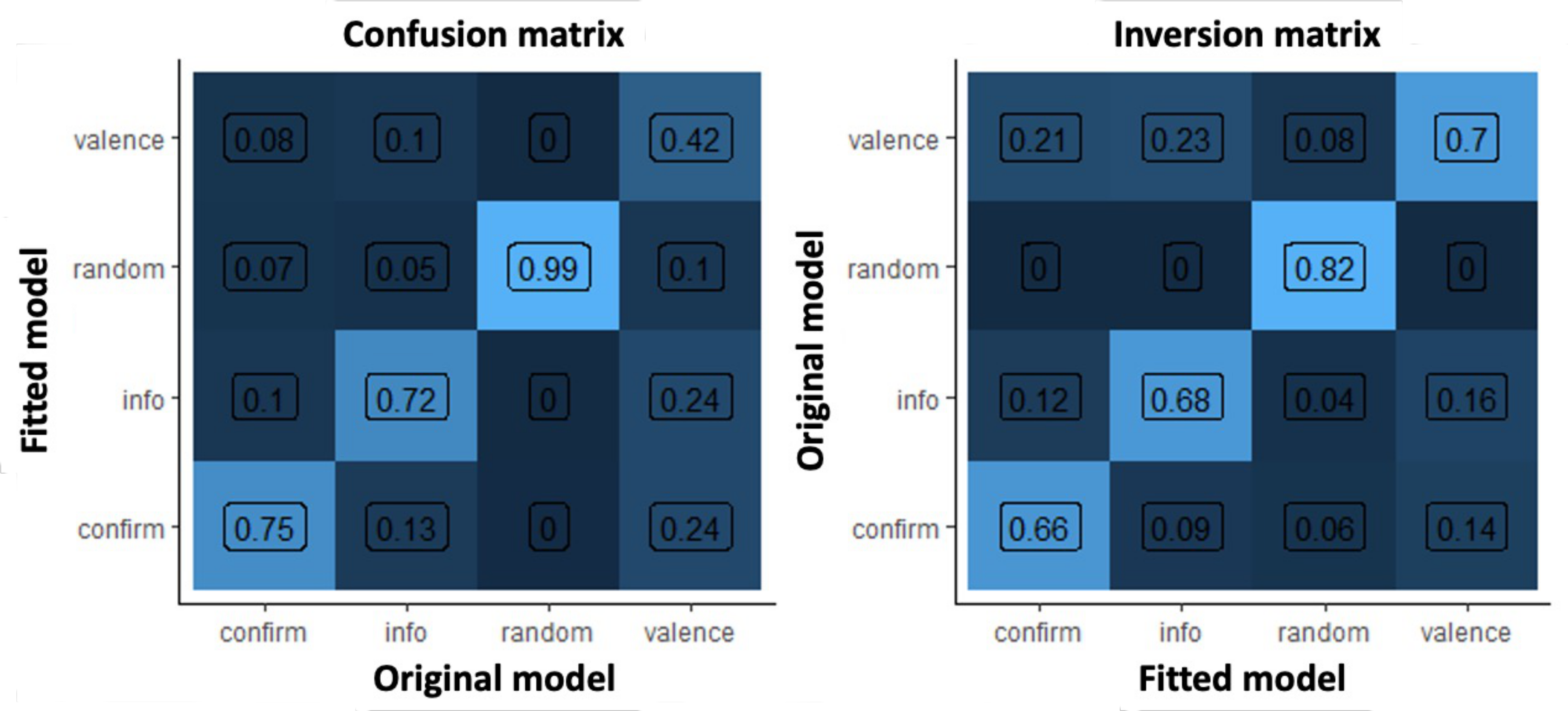

Supplement: Supplementary Figure 9 [file EMS190284-supplement-Supplementary_Figure_9.tiff]

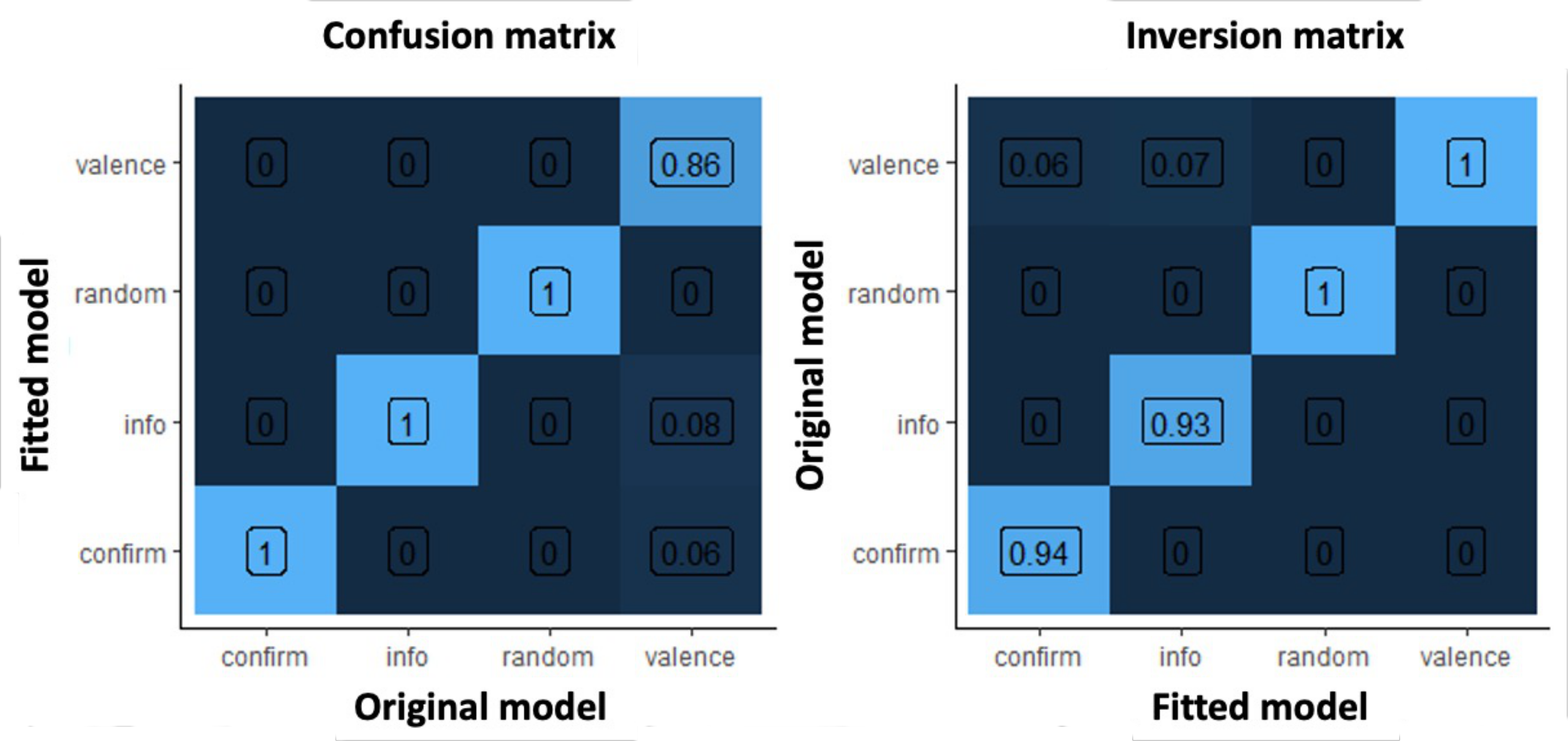

Supplement: Supplementary Figure 10 [file EMS190284-supplement-Supplementary_Figure_10.tiff]

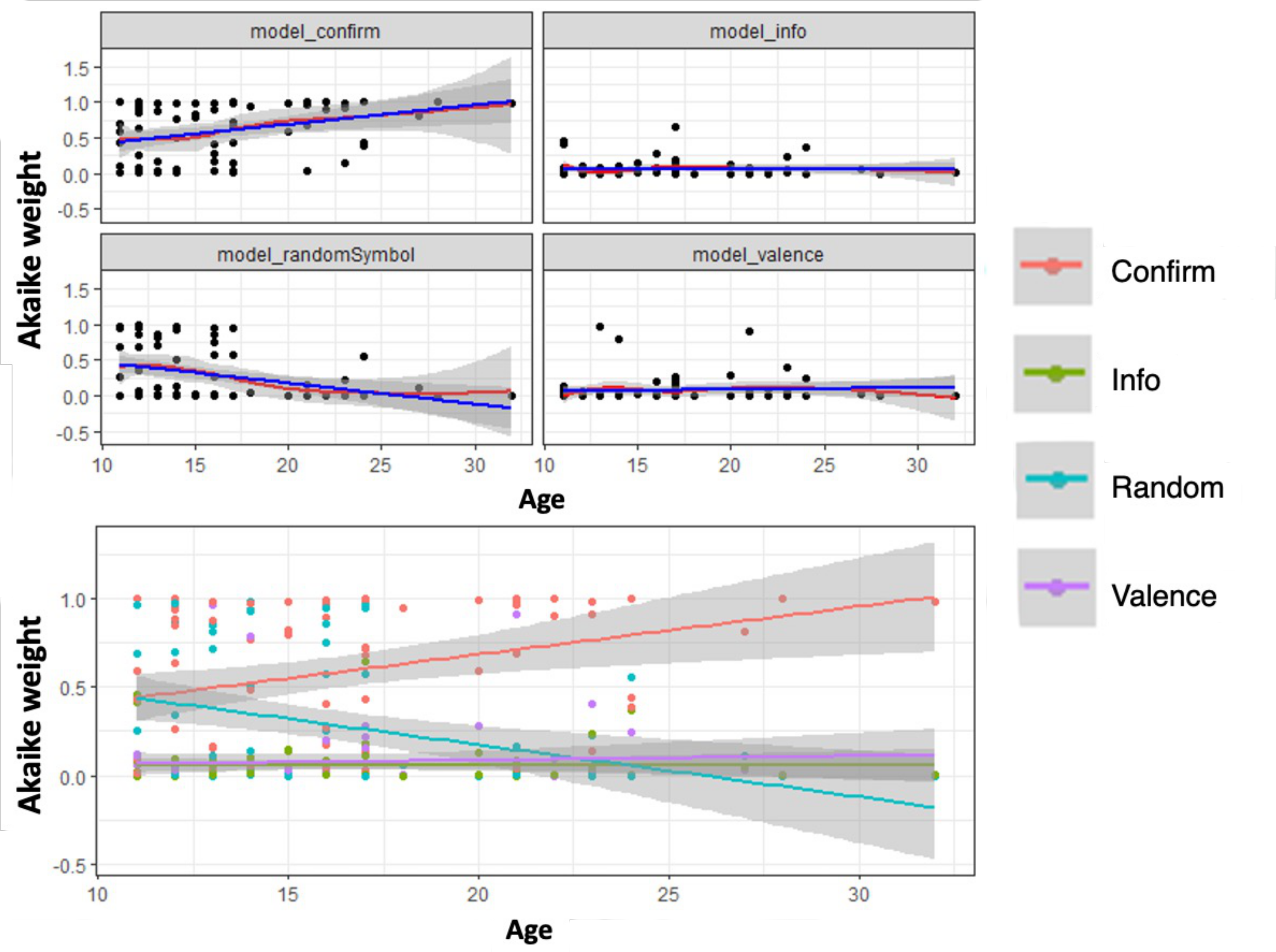

Supplement: Supplementary Figure 11 [file EMS190284-supplement-Supplementary_Figure_11.tiff]

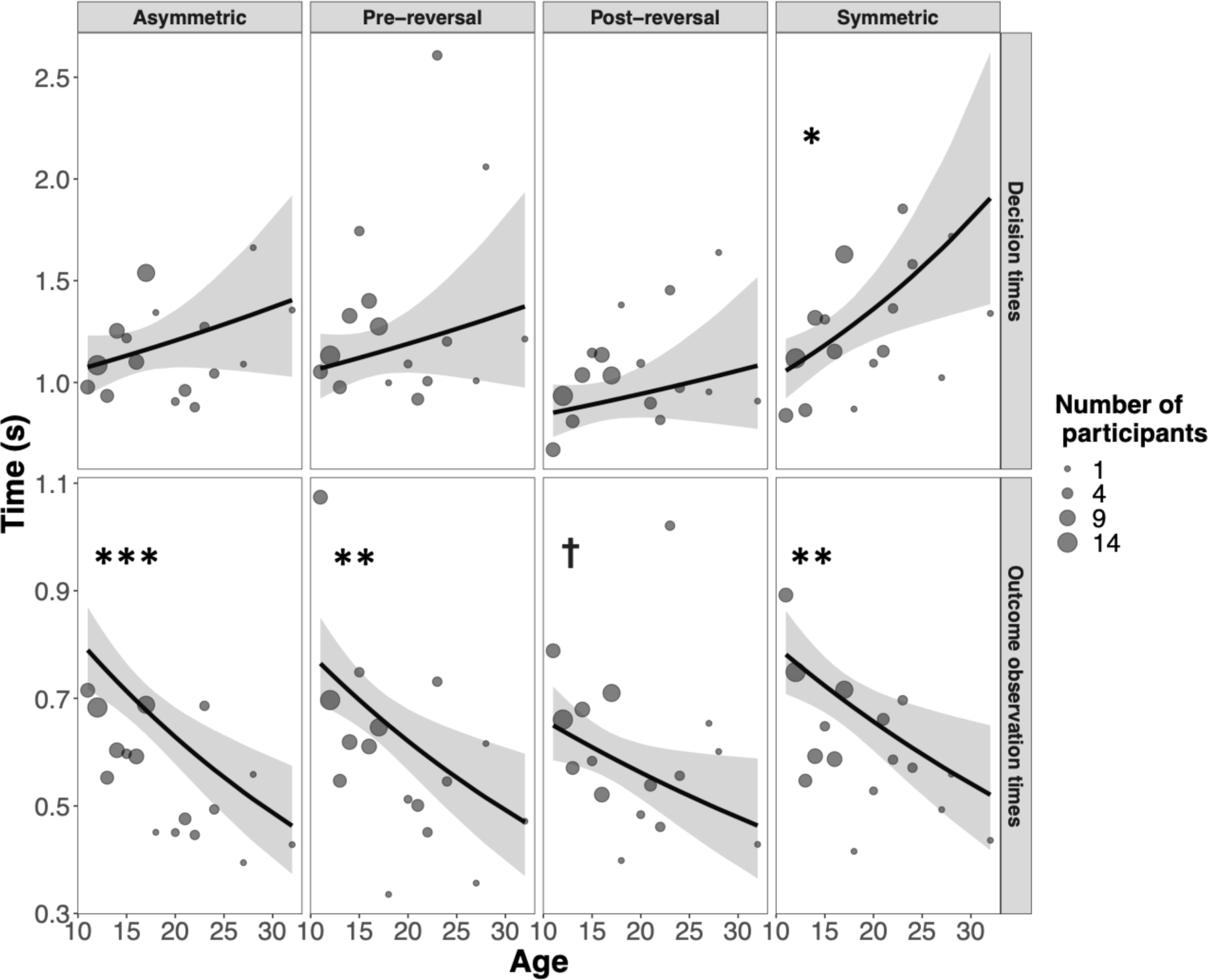

Supplement: Supplementary Figure 12 [file EMS190284-supplement-Supplementary_Figure_12.tiff]
