# Supplementary material for: Confirmatory reinforcement learning changes with age during adolescence
Source: Dev Sci. Author manuscript; Available in PMC 2023 Nov 6. (PMC7615280; doi:10.1111/desc.13330)
Supplement: Supplementary Information [file EMS190284-supplement-Supplementary_Information.docx]

**Supplementary Material**

**Supplementary Material 1: power analysis**

During the revision process, we conducted a power analysis on available data from Palminteri and colleagues (2016). We selected this study because it involved a comparable reinforcement learning task (a two-option reinforcement learning task with stationary and asymmetric reward contingencies) and participants in a similar age range (i.e., 12-32 vs. our 11-32). Re-analyses of the data by Palminteri et al. (2016) showed that the effect size of age on reinforcement learning accuracy (Pearson’s correlation between age and participant-level proportions of correct choices) was moderate (r = 0.3). Power analyses suggested that 82 participants are sufficient to detect this effect size with 80% power, a frequently recommended benchmark (Cohen, 1992). Our sample involved 77 participants, which allows to detect that effect size with 77% power.

**Supplementary Material 2. Behavioural analyses: generalized mixed effect model structure**

*Choice variables*

Given that each of the choice-related dependent variables (DVs) were dichotomous, the three choice-related generalized linear mixed effect models (GLMM^Acc,^ GLMM^Pref^, GLMM^WSLS^) employed a binomial link function. As fixed effects, the choice-related models included a term for participants’ age (standardized integers between 11 and 32), for the trial (i.e., standardized log of integers ranging between 1 and 24 in symmetric and asymmetric conditions, and between 1 and 12 for pre-reversal and post-reversal trials), as well as the interaction between these two terms. The GLMM^Acc^ and GLMM^WSLS^ models also allowed for a 3-way interaction between age, trial and a condition term: a 3-level factor indicating whether trials pertained to the asymmetric, pre-reversal or post-reversal conditions. The GLMM^WSLS^ model additionally included a 3-way interaction between age, trial and the outcome of the previous trial: a 2-level factor indicating whether participants had won or lost on the previous trial. As random effects, all models clustered data at the participant level, using participant IDs as random intercepts. Models also initially included maximal random slopes, that is, all within-subject variables. The GLMM^WSLS^ model resulted in a singular fit, leading us to simplify its random effect structure, as recommended by Barr (2013). Ultimately, this model included one random slope only for the previous outcome term. Terms of the three models are synthesized in Supplementary Table 1.

| **Fixed effects** | **GLMM^Pref^** | **GLMM^Acc^** | **GLMM^WSLS^** |
| --- | --- | --- | --- |
| Age * Trial | X | X | X |
| Age * Trial * Condition |  | X | X |
| Age * Condition * Previous outcome |  |  | X |
| **Random intercepts** |  |  |  |
| Participant ID | X | X | X |
| **Random slopes** |  |  |  |
| Trial | X | X |  |
| Outcome previous trial |  |  | X |
| Trial * Condition |  | X |  |
| ICC | 0.14 | 0.33 | 0.12 |
| Observations | 1833 | 5508 | 6956 |
| Marginal R^2^ | 0.01 | 0.157 | 0.217 |
| Conditional R^2^ | 0.147 | 0.438 | 0.313 |

**Supplementary Table 1. Fixed and random effects probed in generalized mixed effect models (GLMMs) on choice-related variables.** “X” indicates a given term was employed in the model. Empty cells indicate a given term was not employed in the model, either because it was not applicable or because of convergence issues. Interactions included all lower-level main effects and overall trends. Age and trial were standardized to facilitate convergence. Trial was additionally logged to improve fit.

To individuate appropriate functions of the trial term, preliminary analyses compared various fits of a simplified GLMM^Acc^ model including random effects only. These simplified models differed only in the functional form relating trial sequence to accuracy. We compared linear, logarithmic and inverse functions of the trial term. The logarithmic function yielded the best fit (lowest AIC). We then used logarithmic function of the trial term to compare candidate functions of age, this time in the full GLMM^Acc^ model described above. Specifically, we compared linear, quadratic, cubic, logarithmic and inverse functions of age. The linear function of age yielded the best fit. We thus used the logarithmic function of trial and the linear function of age in all remaining modelling (GLMM^Pref^ and GLMM^WSLS^).

*Time variables*

As additional DVs of interest, we ran GLMMs on decision times, GLMM^DT^, and outcome observation times, GLMM^OOT^. Both models employed fixed effects for the trial term, as well as the interaction between age and the condition, a 4-level factor, with levels ‘Asymmetric’, ‘Pre-reversal’, ‘Post-reversal’ and ‘Symmetric’. The GLMM^OOT^ model additionally included the interaction between age and the current choice outcome, a 2-level factor indicating whether participants had won or lost on the current trial. As random effects, both models included participant IDs as random intercepts. As random slopes, due to singular fit or failed convergence of the maximal models (Barr, 2013), the GLMM^DT^ model included the trial and condition term, while the GLMM^OOT^ model included the trial and outcome term. Terms of the three models are synthesized in Supplementary Table 2.

| **Fixed effects** | **GLMM^DT^** | **GLMM^OOT^** |
| --- | --- | --- |
| Trial | X | X |
| Age * Condition | X | X |
| Age * Current outcome |  | X |
| **Random intercepts** |  |  |
| Participant ID | X | X |
| **Random slopes** |  |  |
| Trial | X | X |
| Condition | X |  |
| Previous outcome |  | X |
| ICC | 0.40 | 0.30 |
| Observations | 7341 | 7304 |
| Marginal R^2^ | 0.080 | 0.152 |
| Conditional R^2^ | 0.451 | 0.409 |

**Supplementary Table 2. Fixed and random effects probed in generalized mixed effect models (GLMMs) on time-related variables.** Interactions included all lower-level main effects and overall trends. Age and trial were standardized to facilitate convergence. Trial was additionally logged to improve fit.

**Supplementary Material 3. Non-verbal reasoning**

The matrix reasoning subscale of the WASI (Wechsler, 1999) was also collected after the main task, to be used as an assessment of non-verbal IQ. This data was missing for two participants. In line with previous work (e.g., Chierchia et al., 2019), we found that non-verbal reasoning performance increased with age (Spearman’s ρ = 0.34, p = .003).

Because non-verbal reasoning abilities have been shown to contribute to accuracy in reinforcement learning (e.g., Nussenbaum et al., 2021), we addressed whether the results reported in our study held when controlling for inter-individual differences in non-verbal reasoning. We did this at the behavioural and computational levels.

At the behavioural level, we re-ran each of the models of our study (GLMM^Acc^, GLMM^WSLS^, and GLMM^Pref^) adding non-verbal reasoning as a (z-scored) covariate and allowing this to interact with the same terms that age was allowed to interact with (see Supplementary Material 2 for details). Non-verbal reasoning had no effect on any of the behavioural variables (all ps > .06), except for choosing the preferred option in symmetric trials (χ^2^(1) = 0.417, p = 0.041, slope = 0.04, SE = 0.02). Each of the age-associated patterns reported in the study remained significant (all ps <.003).

At the computational level (Supplementary Fig. 1), non-verbal reasoning was associated with the confirmation model fit (AIC) (ρ = - 0.42, p <.001), as well as inverse temperature (ρ = 0.36, p = .002), but not with confirmation bias (ρ = 0.21, p = .074). This suggests that, similarly to age, non-verbal reasoning abilities were associated with participants’ tendency to adopt a confirmatory learning style and to do so less noisily.


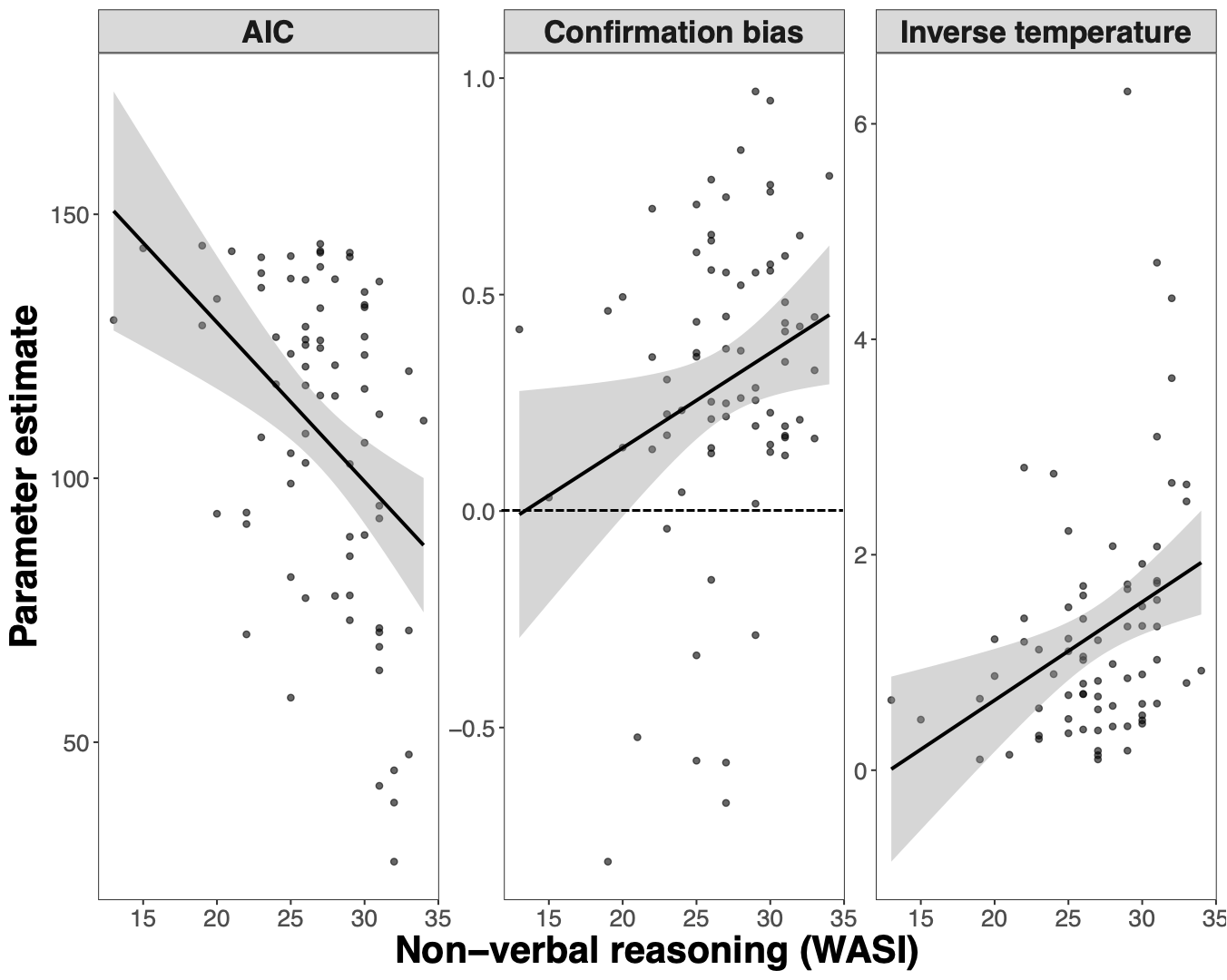


**Supplementary Fig. 1. Model fit (AIC) and parameter estimates of the confirmation model as a function of non-verbal reasoning.** The dashed line in the middle panel indicates potential unbiased values.

In spite of this, multiple regression models continued to show significant trends of age on both AIC (F_(1,72)_ = 11.06, p = .001) and inverse temperature (F_(1,72)_ = 10.69, p = .002). For both of these dependent variables, the variance explained by age (AIC: η_p_^2^  = .20, 90% CI [.08 .33]; inverse temperature: η_p_^2^  = 0.18, 90% CI [0.07, 0.31]) was larger than the variance explained by non-verbal reasoning (AIC: η_p_^2^  = 0.12, 90% CI [0.03, 0.24]; inverse temperature: η_p_^2^  = 0.07, 90% CI [0.01, 0.18]).

Taken together, these results suggest that, while there can be associations between non-verbal reasoning and reinforcement learning (e.g., Nussenbaum et al., 2021), these do not fully account for the age-related associations reported in the study.

**Supplementary Material 4. Age differences in counterfactual updating**

To assess counterfactual learning, we fitted data with a full learning model. This is a model that consists of four learning rates in addition to the inverse temperature parameter. The learning rates are shaped by the following outcomes: 1) wins, 2) foregone wins (i.e., when the unchosen option would have been rewarded), 3) losses and 4) avoided losses (i.e., when the unchosen option would have led to a loss). Having a separate learning rate for all possible conditions allowed us to independently estimate factual and counterfactual learning. As can be seen in the figure below (Supplementary Fig. 2), no age trends were apparent (apart from the age-related increase in inverse temperature, which we also observe in the confirmation model).


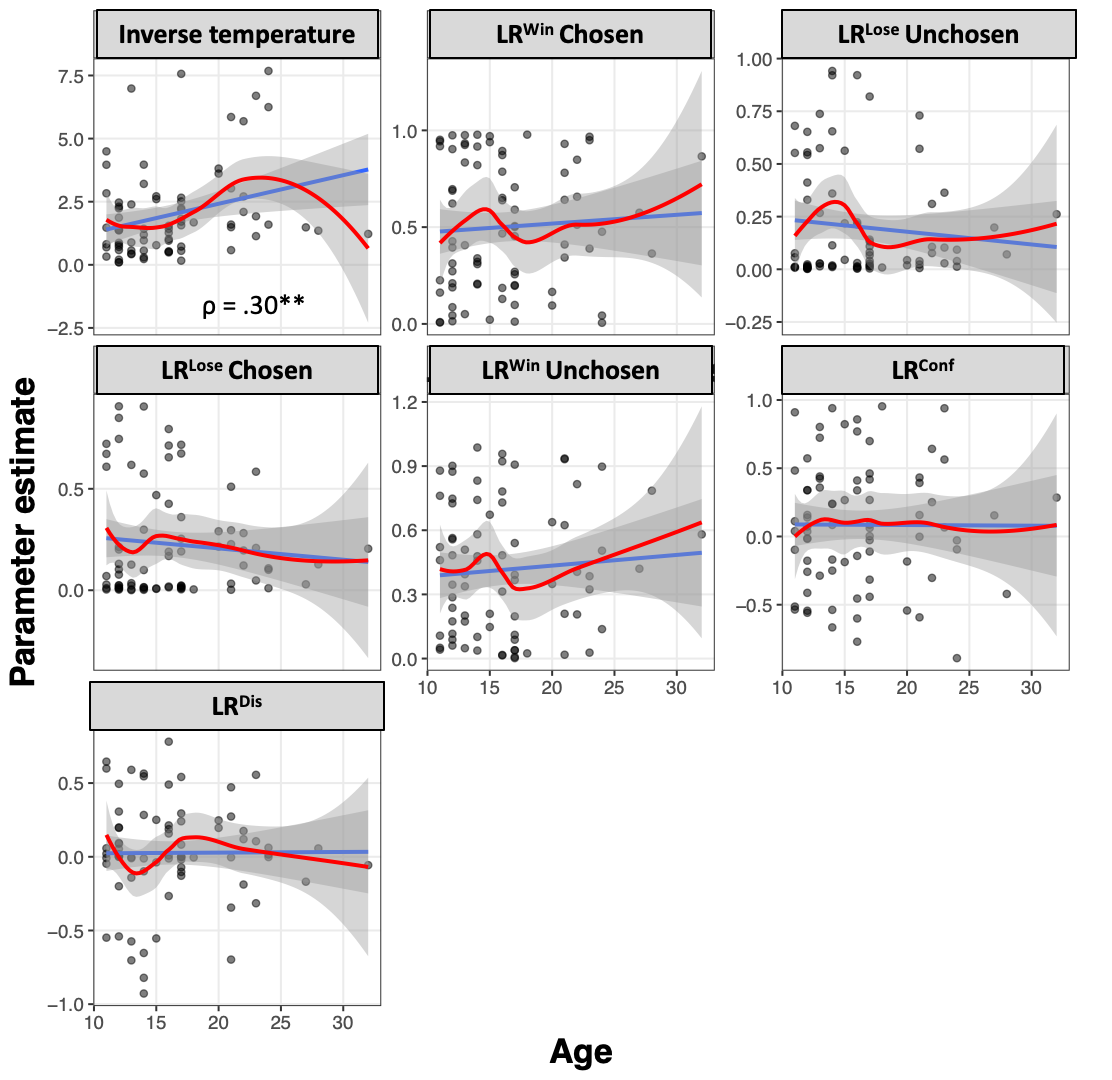


**Supplementary Fig. 2.** Full model parameter estimates and age. *LR^Win^Chosen* = wins, *LR^Lose^Chosen* = losses, *LR^Win^Unchosen* = foregone wins, *LR^Lose^Unchosen* = avoided losses. *LR^Con^* = (*LR^Win^ Chosen - LR^Lose^ Unchosen)*, *LR^Dis^ = (LR^Lose^ Chosen - LR^Win^ Unchosen)*. Blue lines are linear trends, red lines are non-linear (loess fits). In terms of rank correlations, the only significant association is between age and inverse temperature.

**Supplementary Material 5. Parameter estimation at the condition-level**

To establish whether parameter values were significantly different between conditions, we fitted the confirmation model separately for the symmetric, asymmetric and reversal conditions, and compared the fitted parameter values (Supplementary Fig. 3). Three generalized mixed effect models were used to address whether the three estimated parameters varied between conditions and whether these further interacted with age. For inverse temperature, a gamma distribution with log-link function was used, while for *LR^Con^* and *LR^Dis^* the beta family was used. The models revealed a significant main effect of condition on inverse temperature (χ^2^(2) = 14.71, p < .001), due to inverse temperature being lower in asymmetric conditions, relative to both other conditions (ps < .04). In contrast, learning rates did not significantly change between conditions (ps > .20). None of the three models showed significant interactions between age and condition (all ps > 0.34), in line with recent evidence (Nussenbaum et al., 2021). This suggests that the age-related effects did not change as a function of the condition. Indeed, corroborating the main findings of the condition-wide analyses reported in our study, the models revealed a positive linear association between inverse temperature and age (χ^2^(1) = 22.95, p < .001), and no association between age and either of the two learning rates (ps > .11).


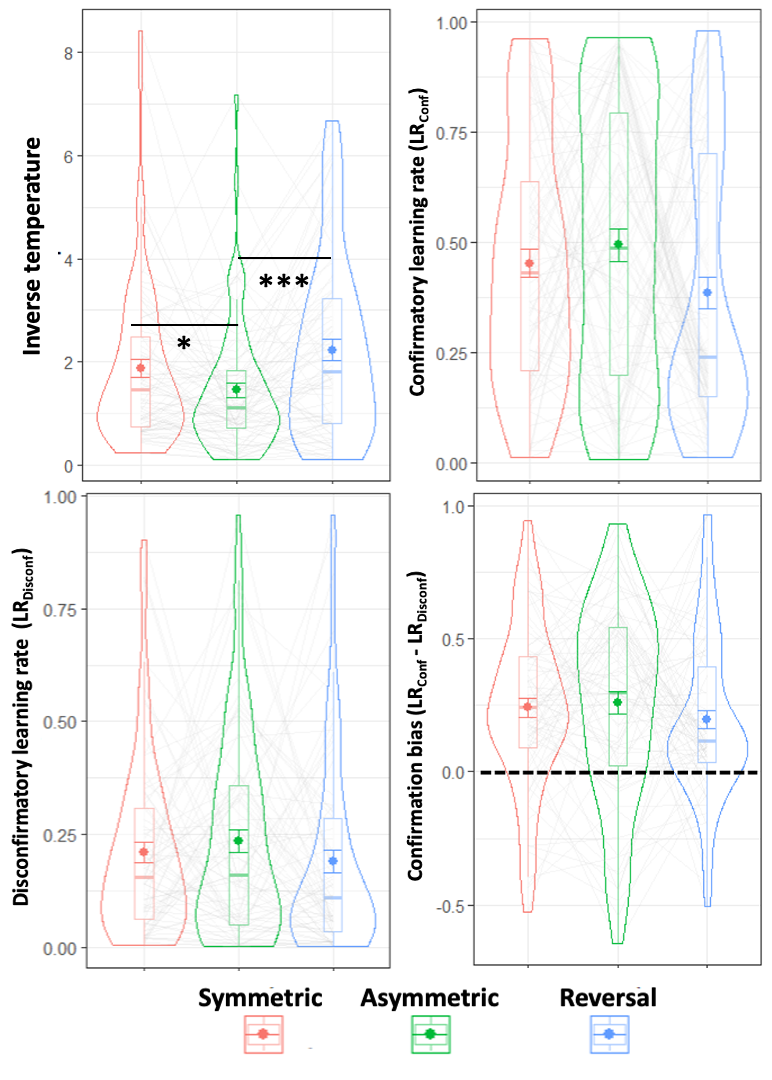


**Supplementary Fig. 3. Condition-level parameter estimates.** Violin plots represent estimated kernel density functions of the data, together with box plots showing the interquartile range, and error bars representing standard errors of the means. The dashed line indicates unbiased learning rates. Mixed models were used to compare the parameter estimates between conditions. ***p<.001, *p<.05 .

**Supplementary Material 6. Interaction between age, condition and trial on accuracy**

Results of a generalized linear mixed effect model on accuracy (GLMM^Acc^) suggested that the positive association between trial and age is reduced in post-reversal trials (Supplementary Fig. 4).


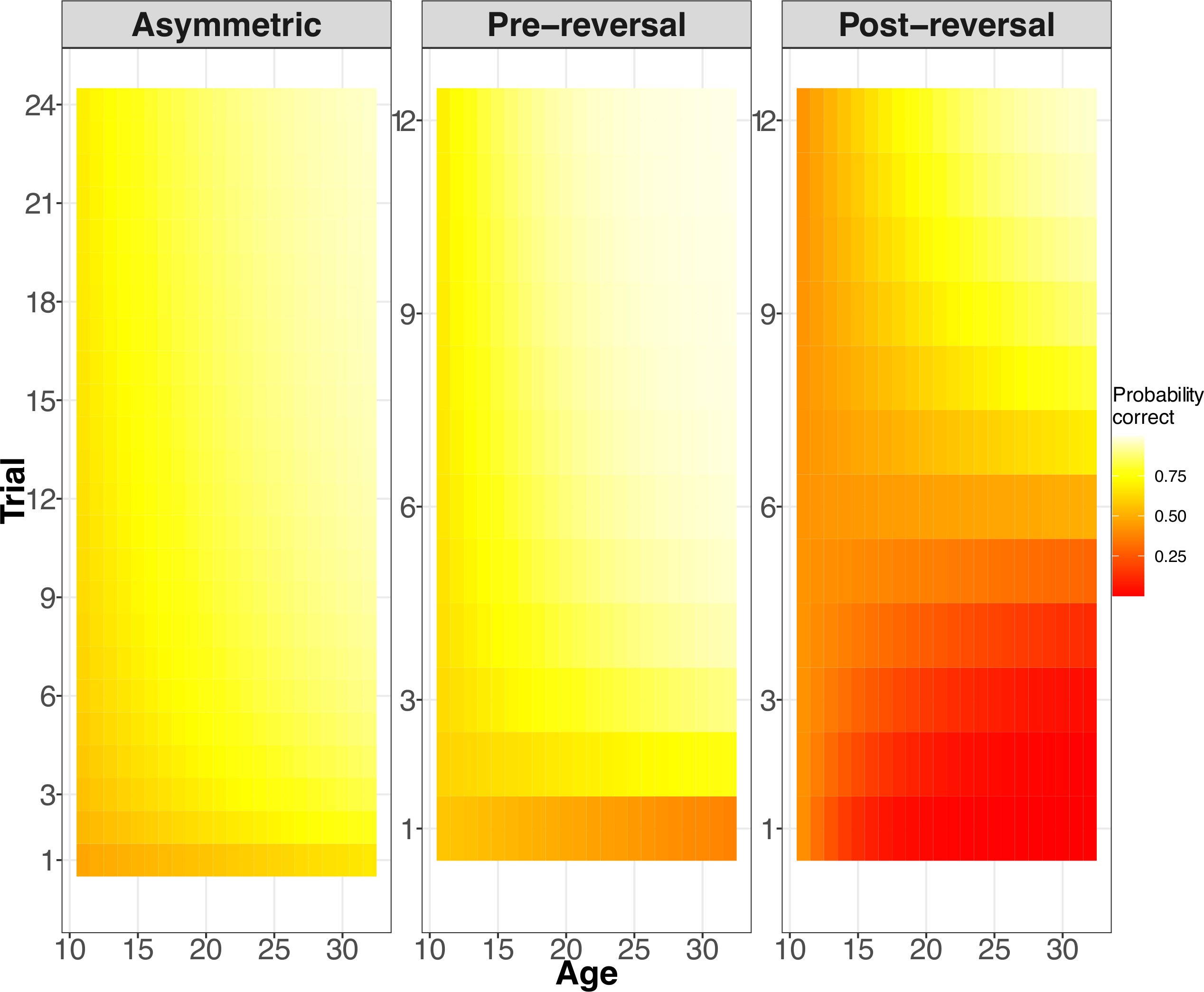


**Supplementary Fig. 4.** Interaction between age (x-axis), trial progression (y-axis) and condition (panels) on accuracy (colour gradient): age-related benefits are momentarily inverted when learning contingencies change in post-reversal trials. Separate models on each pair of conditions showed that the three-way interaction between age, trial, and condition, was significant in comparisons involving post-reversal trials (ps < 0.028), but not the comparison between asymmetric and pre-reversal trials (p = 0.146).

**Supplementary Material 7. Win-stay/lose-shift asymmetry and accuracy**

As illustrated in the introduction of our study, an asymmetry in win-stay/lose-shift (WSLS) behaviour can be a by-product of efficient learning. Given that people become more accurate learners with age, this raises the possibility that the age-related amplification of the asymmetry (reported in the study) might be driven by age-related improvement in learning accuracy. To address this, we conducted several additional exploratory analyses. The first simply involved assessing the amplified WSLS asymmetry in symmetric trials, which are neutral towards accuracy (e.g., there is no way to improve accuracy in these trials). As shown in Supplementary Fig. 5 below, the age-related increase in the win-stay/lose-shift asymmetry was reliably observed in symmetric conditions.


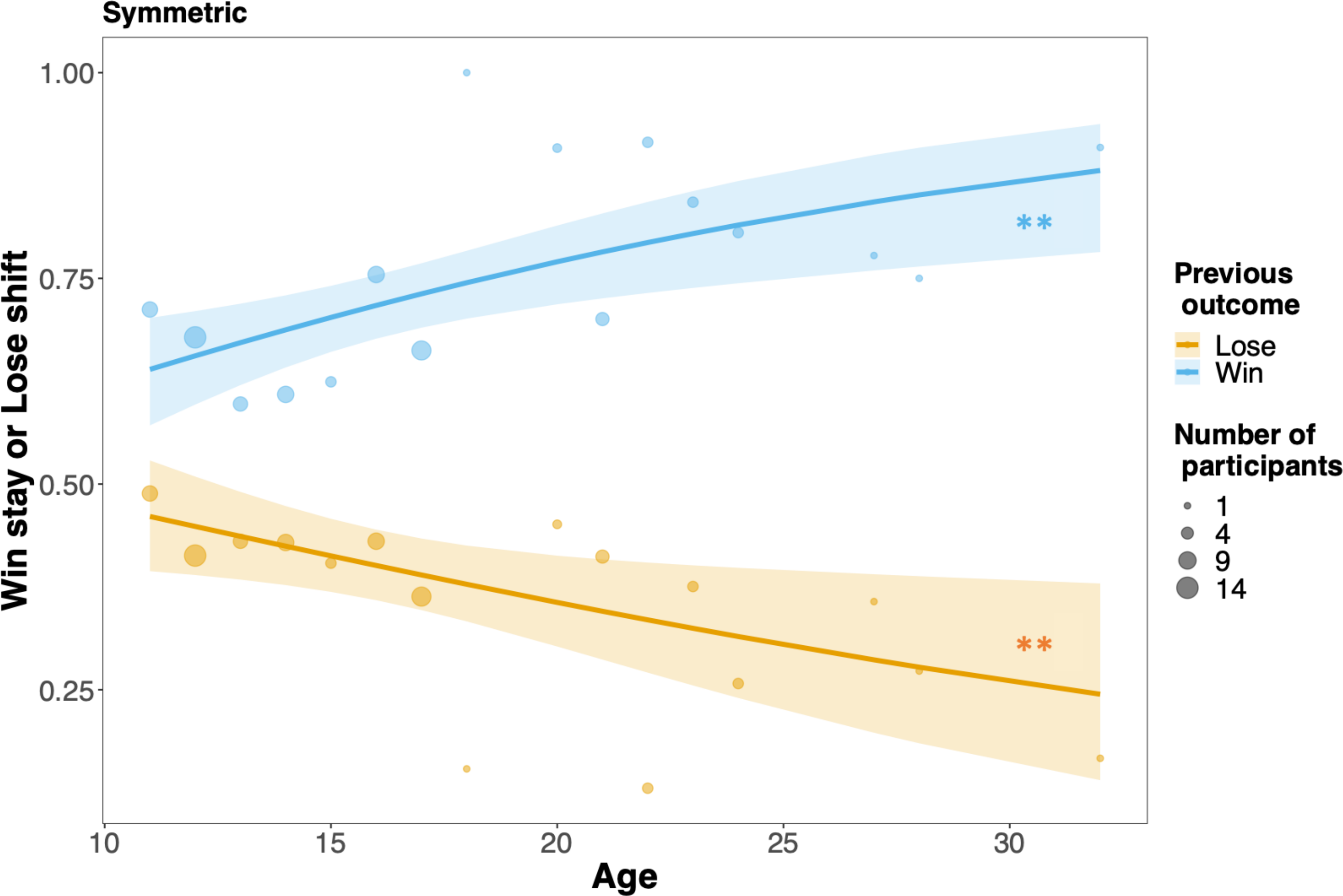
**Supplementary Fig. 5. Win-stay/lose-shift asymmetry is increased with age in symmetric trials only.** Solid lines are estimated fixed effects from generalized mixed models with 95% confidence ribbons. Dots are observed grand means, with surface areas proportional to the number of participants. ** p < .01.

Second, and beyond symmetric trials, to further address whether age-related increases in the WSLS asymmetry are entirely accounted for by accuracy, we asked whether the amplified asymmetry can be detected when controlling for accuracy. There are two possible ways to add an accuracy-related covariate to models assessing the amplified asymmetry. First, we re-ran the WSLS model (GLMM^WSLS^, see Supplementary Material 2) whilst additionally controlling for accuracy at the trial-level. In other words, for each trial in which participants stayed after a win or switched after a loss (as opposed to switched after a win or stayed after a loss), the corresponding trial level predictor of accuracy indicated whether the choice was correct or incorrect. Because this analysis uses a trial-level covariate, it has the possible advantage of more precisely controlling for accuracy. However, the approach has the disadvantage of omitting symmetric trials. In fact, since there is no trial-level measure of accuracy in this condition, symmetric trials were excluded from this analysis. To address this omission, the second approach uses a participant-level, rather than trial-level, covariate of accuracy, by averaging accuracy over all conditions in which accuracy applies. With this we might incur some loss in precision relative to the previous trial-level approach, but it allows symmetric trials to be included into the model. Both models showed clear positive associations between the accuracy term and WSLS (ps < .001), suggesting that accuracy is indeed related to WSLS. However, both models also continued to show robust interactions between age and WSLS (ps < .001) in the same directions as those reported in the manuscript. Taken together, these findings suggests that age related increases in the WSLS asymmetry are not entirely explained by improvements in accuracy.

**Supplementary Material 8. Interaction between age and time variables on accuracy**

To further explore the relationship between the time variables, age and accuracy, we ran two further models: the first asked whether age and decision times interacted to modulate accuracy (GLMM^Acc-DT^). The second asked whether age and outcome observation times interacted to modulate accuracy (GLMM^Acc-OOT^). As for the previous accuracy model (GLMM^Acc^), both models focussed on the conditions in which accuracy is applicable (namely, asymmetric and reversal conditions) and modelled the binary dependent variable (correct or incorrect) with a binomial link function. As fixed effect predictors, both models allowed the respective time variable (decision times in GLMM^Acc-DT^ and outcome observation times of the previous trial of the same condition in GLMM^Acc-OOT^) to interact with age. As per random effects, both models used participant IDs as random intercepts and the respective time variables as random slopes. The results of the modelling are plotted in Supplementary Fig. 6, below.


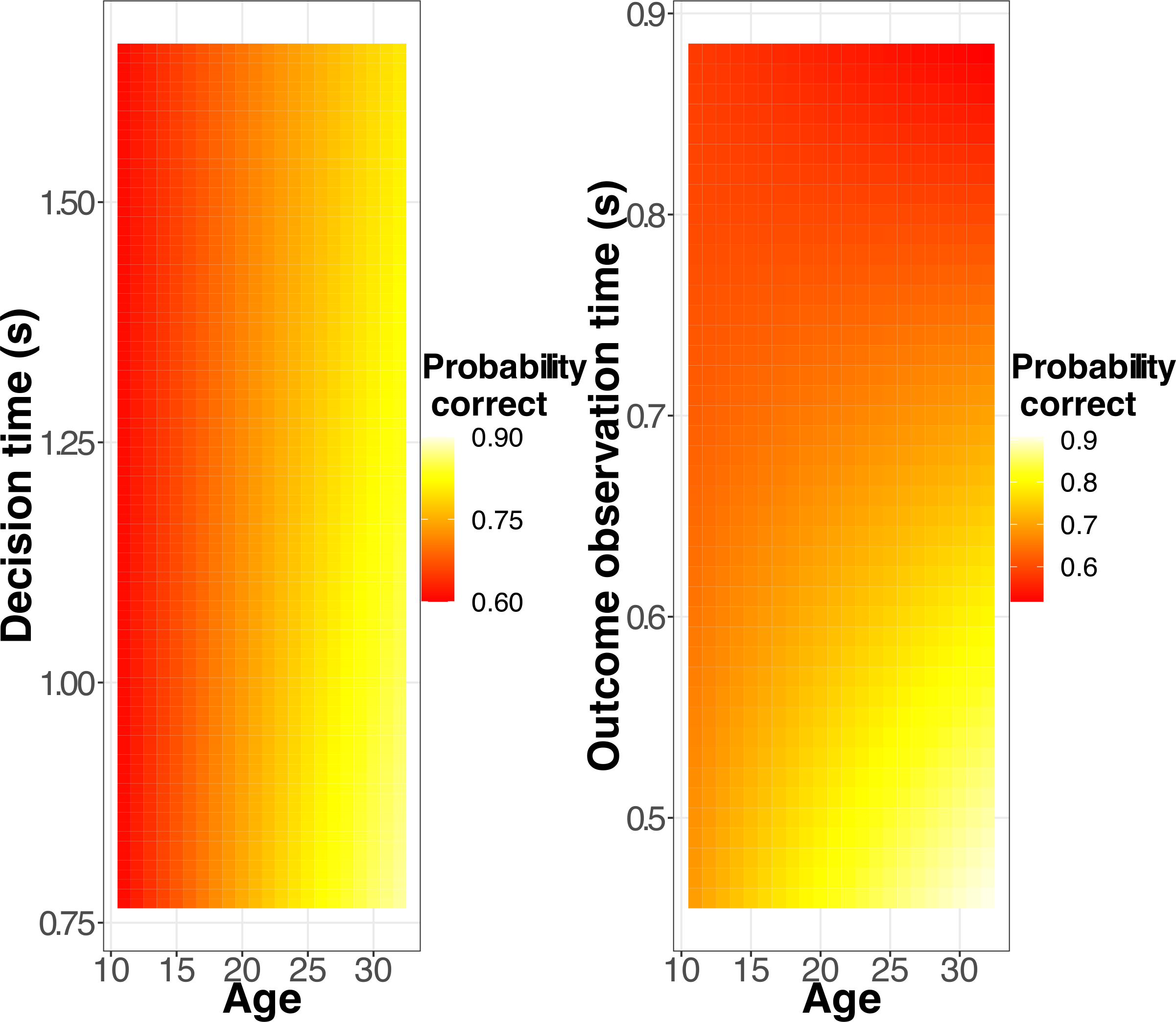


**Supplementary Fig. 6. Interaction between age (x-axis) and time variables (y-axis) in modulating accuracy (colour gradient).** Accuracy was associated with shorter decision and outcome observation times, and this association became stronger with age.

**Supplementary Material 9. Model and Parameter Recovery**

*Parameter recovery*

To estimate how well the winning model (i.e. the confirmation model) recovers the true parameter values, we simulated 1000 individual data sets using parameter values that were sampled from a uniform distribution whose minimum and maximum were set to 89% HDI of the parameter values fitted to the participant data. We found a 65% correlation between the original and recovered inverse temperature parameter, 71% correlation between the confirmatory learning rates (*LR^Co^*^n^) and 71% correlation between the disconfirmatory learning rates (*LR^Dis^*). All correlations were significant (all p < .001, Pearson’s) (Supplementary Fig. 7).


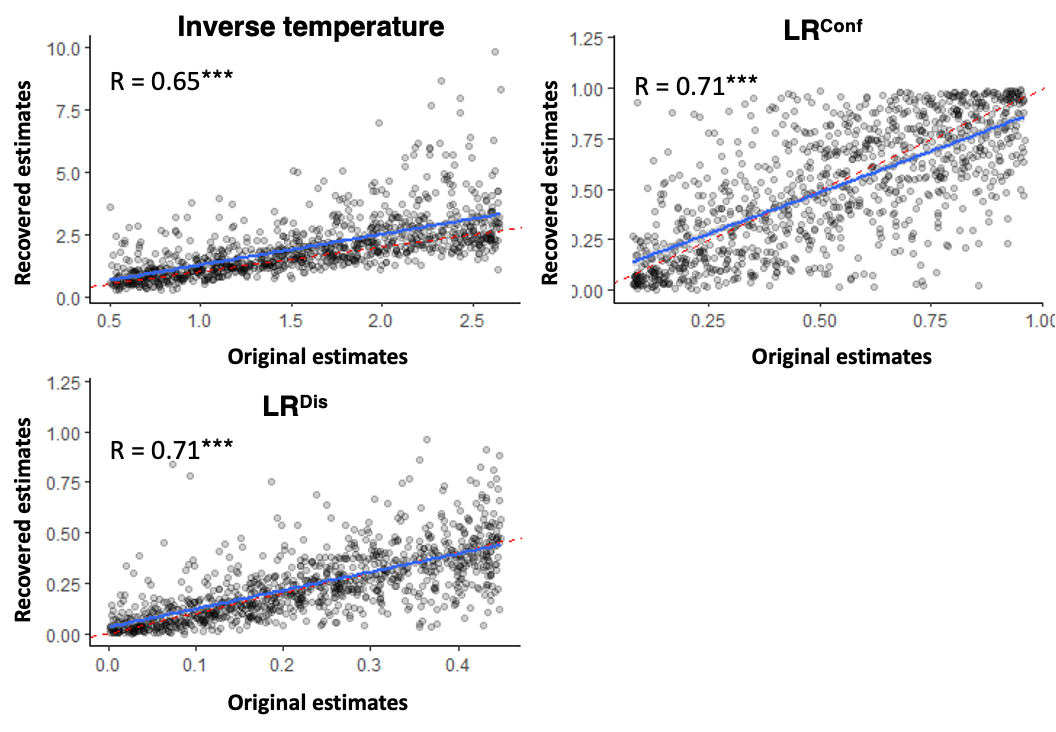


**Supplementary Fig. 7. Confirmation model parameter recovery.** Blue line represents an average, red dashed line is the identity line (perfect recovery). R is Pearson’s correlation coefficient.

*Model recovery*

For the model recovery, we followed a similar procedure as above. For each model (i.e., confirmation, valence, information and random), we first simulated 1000 individual data sets using parameter values sampled from a uniform distribution. The minimum and maximum of the distribution were set to 89% HDI of fitted parameter values. Each data set was then fitted with each model. We established the best fitting model for each simulated data set by comparing the AIC values and constructed a confusion matrix and an inversion matrix. The confusion matrix shows how frequently a given model was the best fit to data generated from each of the four models. The inversion matrix shows which models are likely to be the true model if a given model was selected as the best. We did this both on the individual level, using simple AIC comparison, and on the group level (50 groups of 20 data sets), using Bayesian model selection based on AIC weights (the same approach employed elsewhere in our study). The individual-level approach allowed us to estimate how often the best fitting model was correctly selected for each participant. The group-level approach allowed us to check whether the errors on the individual level were likely to affect the model selection for the whole group.

Overall, the individual-level model recovery approach showed lower recoverability levels than expected (Supplementary Fig. 8). This suggests that some participants might have used a different learning strategy than the one assigned by the model selection. For example, some participants might have used a valence model instead of a confirmation model and vice versa.


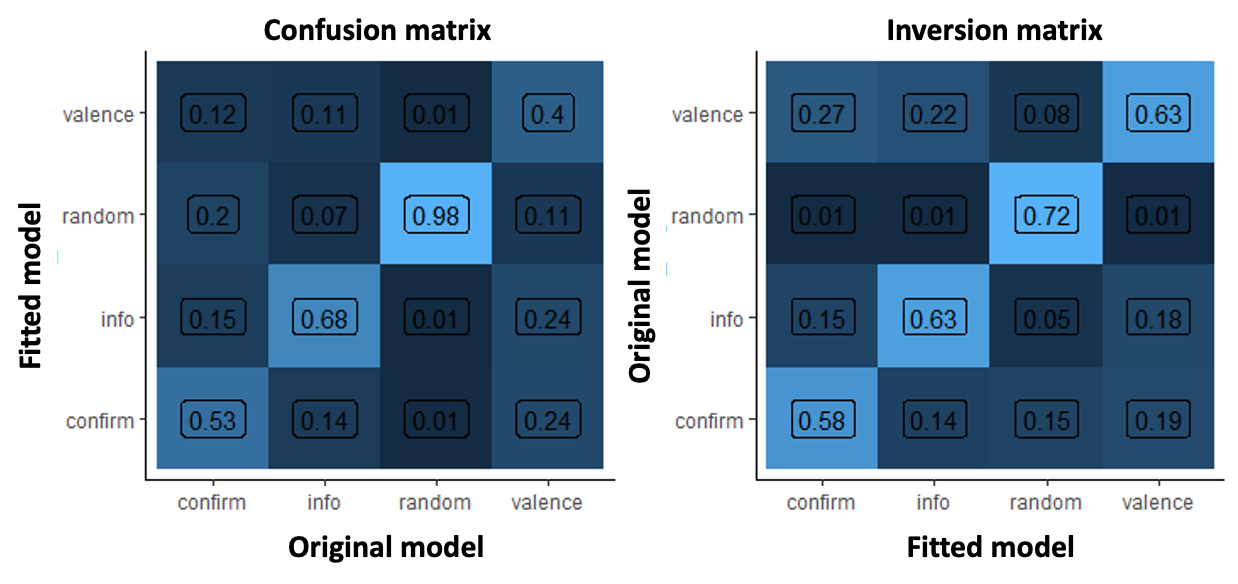


**Supplementary Fig. 8. Individual model recovery.** Data generated from participants’ estimated parameter values.

This was likely due to a relatively high level of noise in the participants’ data, rather than the models themselves. Indeed, as there were quite a few participants whose behaviour was close to random, it is likely that the new data sets that were generated based on that behaviour mirrored this noise. To test this, we repeated the individual model recovery with different data sets that were generated using a wider range of parameter values (i.e., beyond the ones that were observed). Specifically, these data sets were generated by drawing beta (i.e., inverse temperature) from a gamma distribution with shape = 1.2 and scale =5, while learning rates were drawn from a beta distribution with shape1 = 1.1 and shape2 = 1.1. As shown below, these data sets led to a better model recovery (Supplementary Fig. 9). This suggests that the original model recovery was indeed impeded by participants’ noisiness (i.e., inverse temperature), rather than by misspecified models.


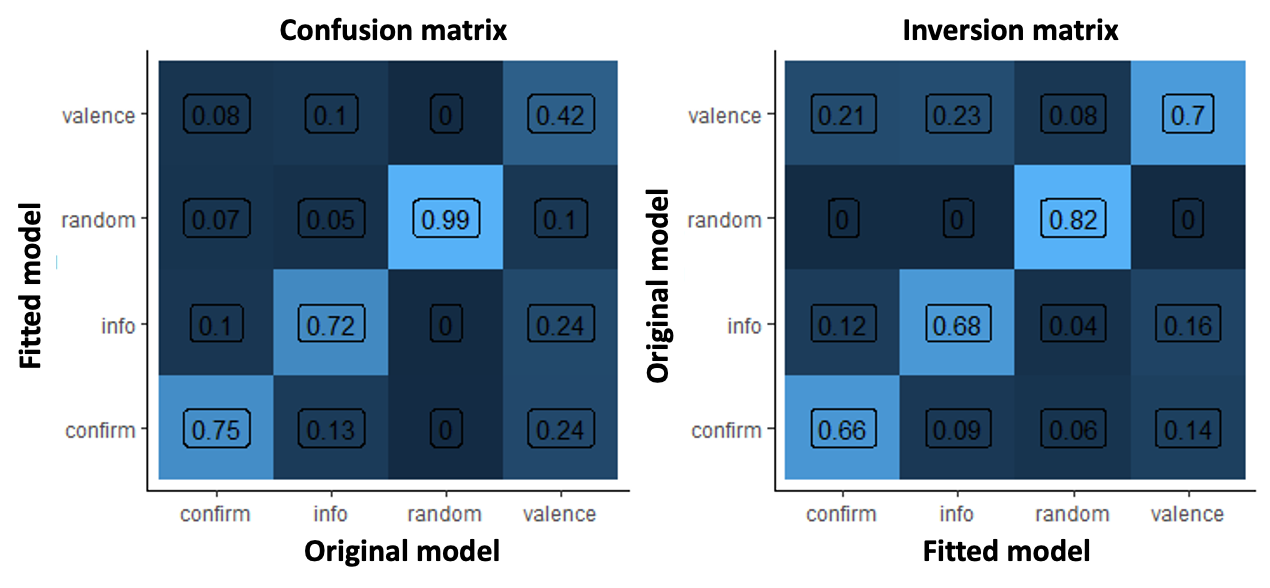


**Supplementary Fig. 9. Individual model recovery.** Data generated from a wider range of possible parameter values.

The group level recovery, on the other hand, was excellent. suggesting that while the model selection might have misassigned some of the participants, it is highly likely that the overall winning model, that is the confirmation model, was assigned correctly (Supplementary Fig. 10.


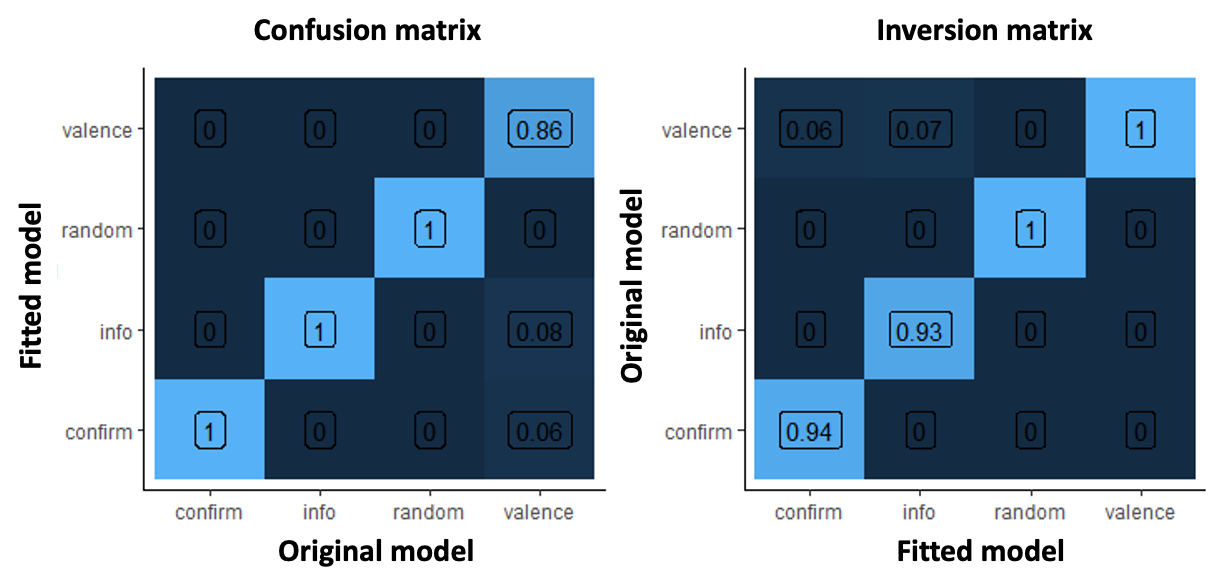


**Supplementary Fig. 10. Group Model Recovery.** Data generated from participants’ parameter values. Data sets were grouped into groups of 20 datasets and underwent Bayesian model selection.

**Supplementary Material 10. Best fitting model distribution across age**

To assess whether the best fitting model changes over age we assessed the relationship between Akaike weights, a measure of relative model fit, and age. The plot below (Supplementary Fig. 11), suggests that while the behaviour of the youngest participants was similarly described by the random model and the confirmation model, the relative discrepancy between these models increased with age.

This was statistically assessed by a running mixed model with AIC values as the dependent variable, and age and the computational model as the independent variables (as well as 1 random intercept for each participant). The model confirmed that the relative discrepancy between the random and confirmation models increased significantly with age (slope_confirm – random_ = -8.06, p < .001, Tukey-corrected).


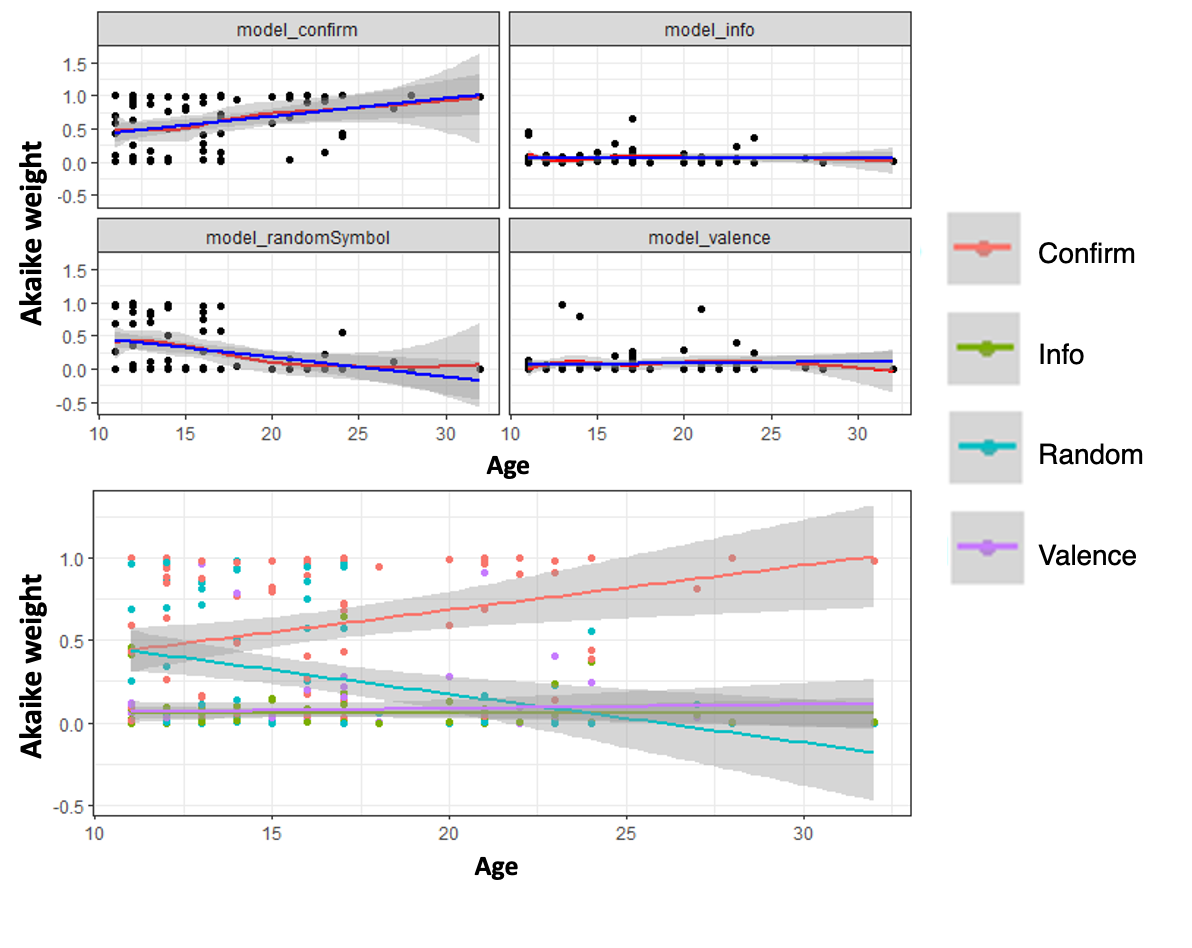
**Supplementary Fig. 11. Akaike weights for all fitted models across age.** Top: each model is plotted separately. Blue lines are linear trends, red lines are non-linear (loess fits). Bottom: models are plotted together, and lines are linear trends.

**Supplementary Material 11. Time variables by condition**

There was no association between age and decision times, except for in the symmetric condition (Supplementary Fig. 12).

**
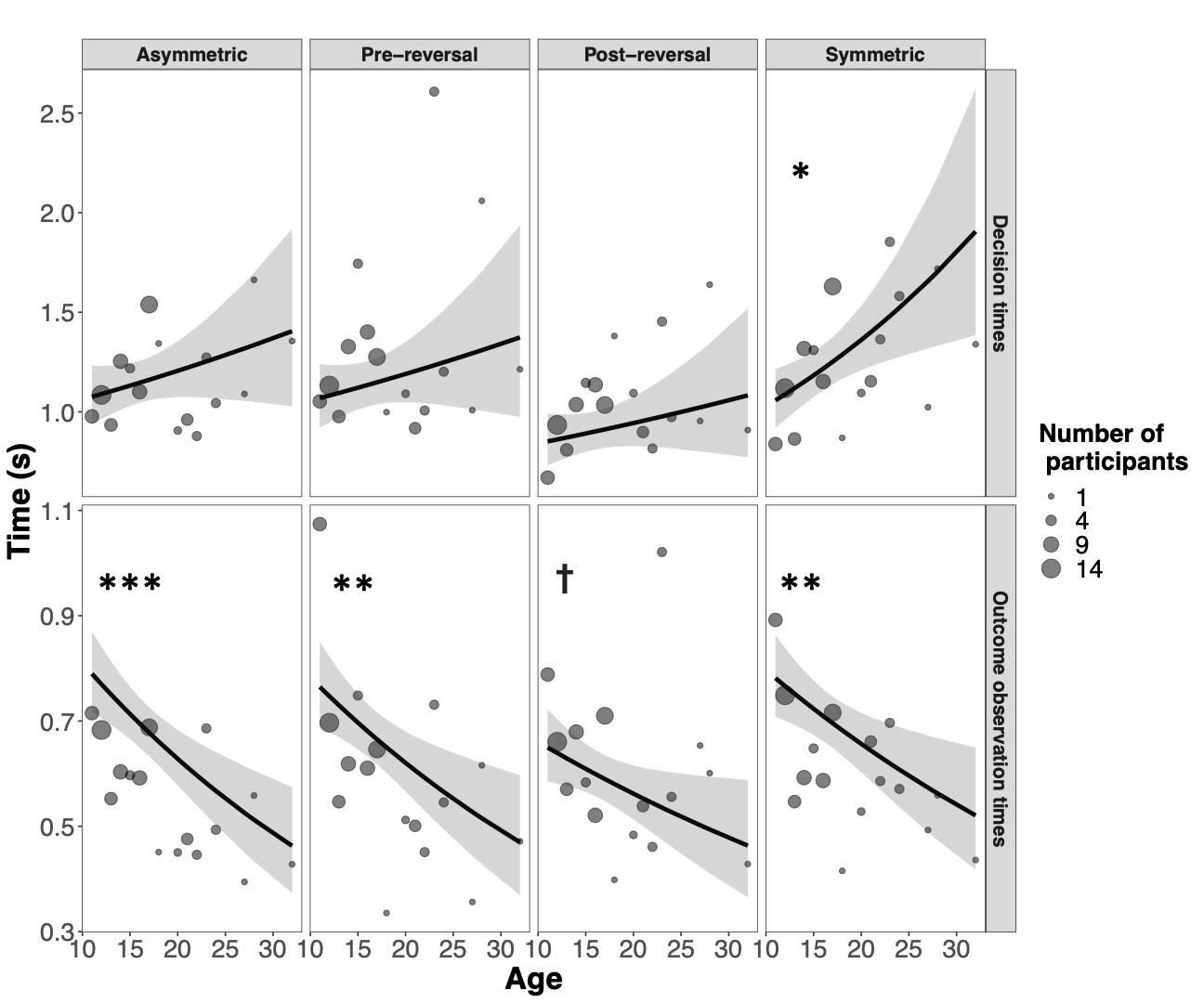
**

**Supplementary Fig. 12. Time variables by condition.** Exploratory condition-level associations between age and two time-related variables: decision times and outcome observation times. Solid lines are estimated fixed effects from generalized mixed models with 95% confidence ribbons. Circles are observed grand medians. P-values are Bonferroni corrected for four comparisons. *** p < .001, ** p < .01, *p < .05, † p <.10.

**References**

Chierchia, G., Fuhrmann, D., Knoll, L. J., Pi-Sunyer, B. P., Sakhardande, A. L., & Blakemore, S.-J. (2019). The matrix reasoning item bank (MaRs-IB): novel, open-access abstract reasoning items for adolescents and adults. *Royal Society Open Science*, *6*(10), 190232. https://doi.org/10.1098/rsos.190232

Nussenbaum, K., Velez, J., Washington, B., & Hamling, H. (2021). *Flexibility in valenced reinforcement learning computations across development*. https://psyarxiv.com/5f9uc
